# Independent Evolutionary Origin of *fem* Paralogous Genes and Complementary Sex Determination in Hymenopteran Insects

**DOI:** 10.1371/journal.pone.0091883

**Published:** 2014-04-17

**Authors:** Vasco Koch, Inga Nissen, Björn D. Schmitt, Martin Beye

**Affiliations:** Institute of Evolutionary Genetics, Heinrich Heine University Duesseldorf, Duesseldorf, Germany; University of Sussex, United Kingdom

## Abstract

The primary signal of sex determination in the honeybee, the *complementary sex determiner* (*csd*) gene, evolved from a gene duplication event from an ancestral copy of the *fem* gene. Recently, other paralogs of the *fem* gene have been identified in several ant and bumblebee genomes. This discovery and the close phylogenetic relationship of the paralogous gene sequences led to the hypothesis of a single ancestry of the *csd* genetic system of complementary sex determination in the Hymenopteran insects, in which the *fem* and *csd* gene copies evolved as a unit in concert with the mutual transfers of sequences (concerted evolution). Here, we show that the paralogous gene copies evolved repeatedly through independent gene duplication events in the honeybee, bumblebee, and ant lineage. We detected no sequence tracts that would indicate a DNA transfer between the *fem* and the *fem1/csd* genes between different ant and bee species. Instead, we found tracts of duplication events in other genomic locations, suggesting that gene duplication was a frequent event in the evolution of these genes. These and other evidences suggest that the *fem1/csd* gene originated repeatedly through gene duplications in the bumblebee, honeybee, and ant lineages in the last 100 million years. Signatures of concerted evolution were not detectable, implicating that the gene tree based on neutral synonymous sites represents the phylogenetic relationships and origins of the *fem* and *fem1/csd* genes. Our results further imply that the *fem1* and *csd* gene in bumblebees, honeybees, and ants are not orthologs, because they originated independently from the *fem* gene. Hence, the widely shared and conserved complementary sex determination mechanism in Hymenopteran insects is controlled by different genes and molecular processes. These findings highlight the limits of comparative genomics and emphasize the requirement to study gene functions in different species and major hymenopteran lineages.

## Introduction

Complementary sex determination, in which the heterozygous genotype at a certain locus determines femaleness, is widely shared in hymenopteran insects and has a deep ancestry [1], [2]. Thus far, the underlying gene *complementary sex determiner* (*csd*) has been identified in the western honeybee (*Apis mellifera*) by positional cloning and knockdown studies [3], [4]. The *csd* gene encodes an SR-type protein. Csd proteins derived from the heterozygous *csd* genotype induce the female sex pathway by directing the female splicing of the primary transcripts of the *fem* gene [4], [5]. The resulting female mRNAs subsequently encode the functional Fem proteins. Csd proteins derived from the hemizygous or homozygous genotypes are not required for sex determination. The male splicing of the *fem* transcripts results by default. The male-specific exons contain a translational stop codon to prematurely stop translation. The absence of functional Fem proteins leads to the development of maleness [5]. More than 14 *csd* alleles have been identified in local honeybee populations, which show an average of 3% pairwise difference in their entire amino acid encoding sequence [6], [7].

The low divergence of the honeybee *csd* and *fem* genes at synonymous sites compared to bumblebee and stingless bee sequences suggests that the *csd* gene was derived from a gene duplication event of an ancestral copy of the *fem* gene in the honeybee lineage [4]. The *csd* gene was shaped by positive selection shortly after it originated [4], [8]. *fem* is the putative ortholog of the *transformer* (*tra*) gene [4], a key sex-determining gene in *Drosophila melanogaster*. However, unambiguous homology relies on identities in a 30-amino-acid motif deduced from another dipteran ortholog of the *tra* gene from *Ceratitis capitata* [9], [10].

A recent study found repeated duplicates of the *fem* gene in four ant and two bumblebee genomes [11]. The transcripts of these *fem* genes are sex-specifically spliced, suggesting a conserved sex-determining role of this gene. The function of the duplicated copies are thus far unknown [11]. The wasp *Nasonia vitripennis*, however, lacks a sister copy of the *fem*/*tra* gene [12]. In this study, we named the other copies of the *fem* gene *fem1*. This is because we have no functional information as to whether these genes control the complementary sex determination process as in honeybees.

**Figure 1:**
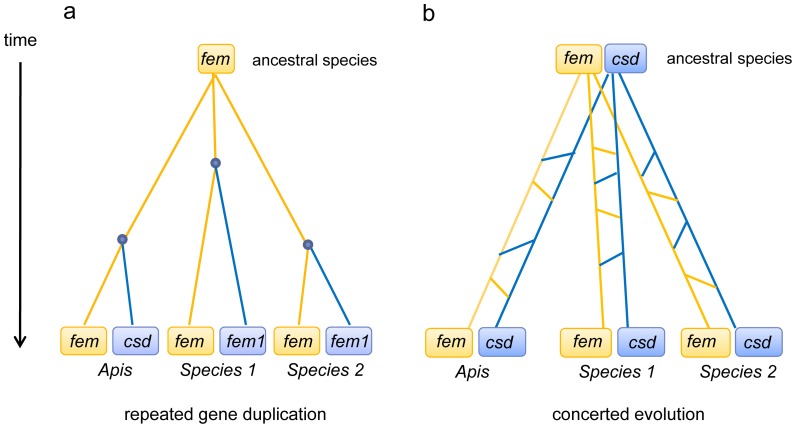
Two models for the evolutionary history of *fem* paralogous genes in ants and bees: (a) repeated gene duplication and (b) concerted evolution. Points in (**a**) denote gene duplication events giving rise to two gene copies. Connecting lines in (**b**) between branches indicate concerted evolution events resulting from unequal crossing over and/or gene conversion.

The phylogenetic relationships deduced from coding nucleotide sequences [11] showed that the paralogous gene pairs of *fem* and *csd/fem1* are more closely related in four ant species, the bumblebee and, as previously shown, the honeybee lineage. Figure S1 shows the sequence relationship of the genes for the neutral synonymous sites. Two recent studies [11], [13] have proposed that in contrast to a model of independent gene duplications, the most parsimonious explanation of the close relationship between the *fem* and *fem1/csd* sequences is that concerted evolution (either due to repeated unequal crossing-over or gene conversion) homogenized the duplicated copies in the different lineages (Fig. 1). One or a few ancestral duplication events gave rise to the *csd* gene and complementary sex determination observed in the Hymenoptera order [11], [13]. The process of concerted evolution between the *fem* and *fem1/csd* genes repeatedly homogenized the two loci, producing the low divergence in the gene pair that we find today.

Here, we readdress the question of whether the *fem1/csd* copies repeatedly evolved through gene duplication (Fig. 1a) or whether the *fem* and *fem1/csd* gene pairs evolved through concerted evolution (Fig. 1b). The clarification of this question will provide fruitful insight into the evolution of paralogous genes and the evolution of a complementary sex determination system.

The arguments given below prompted us to further investigate this question.

1. Studies at the genome-wide scale showed that concerted evolution only affects 2% of the paralogous gene pairs [14], suggesting that this process rarely acts as a homogenizing force between paralogs.
2. The rate for the rise of new paralogous gene copies is 0.01 per gene per million years [15], [16]. This suggests that new duplicates of the *fem* gene can repeatedly originate in different hymenopteran lineages, which have an evolutionary history of more than 120 million years [17]–[20].
3. The evidence for concerted evolution between the paralogs provided thus far, namely, (i) the alternative tree topologies of the *fem* and *fem1*/*csd* nucleotide sequences [13] and (ii) the putative gene conversion tracts in the nucleotide sequence [11], [13], could also result from a heterogeneity in the sequence divergence, a recombination event between the *csd* alleles, methodological problems or homoplasic (convergent) nucleotide changes [4], [6], [8], [21].

Here, we present evidence suggesting that the paralogous gene copies *fem1*/*csd* in ants, bumblebees and honeybees evolved independently and repeatedly through a series of gene duplication events (Fig. 1a).

## Results

### Amino acid changes in the MRCA ancestral sequences of bees and ants are shared between the Fem and Csd/Fem1 proteins

**Figure 2:**
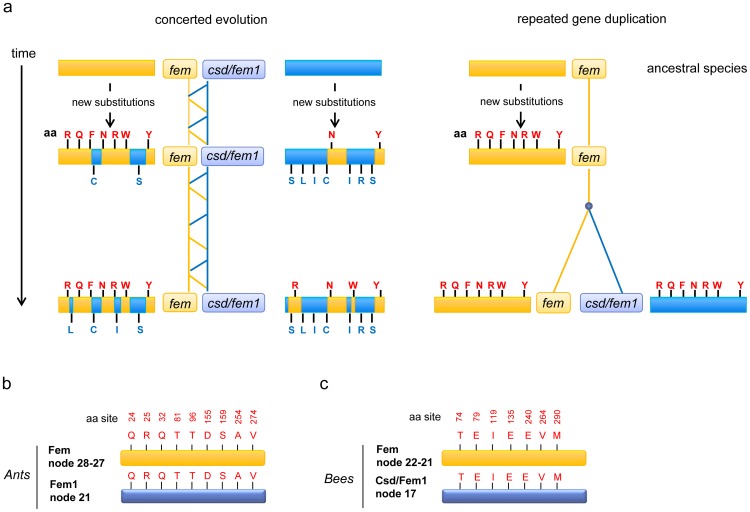
The evolutionary fate of *fem* gene substitutions in an evolutionary window predating the putative repeated gene duplications. (**a**) The expected evolutionary fate of *fem* substitutions in the paralogous genes *fem* and *csd*/*fem1* under the model of concerted evolution and repeated gene duplication. (**b, c**) The letters above the yellow boxes show the inferred amino acid changes in the Fem protein tree that evolved during the evolutionary window of the MRCA of ants and bees and the MRCAs of ants (**b**) and of bees (**c**). Letters above the blue boxes indicate the amino acid residues that are found at the homologous sites in the ancestral Csd/Fem1 protein sequence of the MRCA of ants (**b**) and of bees (**c**). Numbers above the letters designate the homologous sites in the Fem amino acid sequence alignment. Numbers before the boxes indicate the nodes (Fig. S2) used to infer the ancestral sequence information. aa denotes amino acid.

To find further support for either the repeated gene duplication model or the concerted evolution model, we followed the evolutionary trajectory of substitutions that led to amino acid changes in the ancestral sequences of the most recent common ancestor (MRCA) of bees and ants (Fig. 2a). This evolutionary window predates the timing of the different gene duplication events under the repeated duplication model and can therefore provide unique information about the evolutionary history of the sister copies. Under the concerted evolution model, we would expect to find unique substitutions in the ancestral sequences of the MRCA of ants and bees, which are confined to the *fem* or the *csd/fem1* gene (Fig. 2a). This pattern would arise because the two sister copies originated only once in Hymenoptera [11]and accumulated substitutions separately due to their separate evolutionary history, which predates the MRCA of bees and ants [11]. Concerted evolution, the exchange of sequences between evolutionary old paralogous genes, would partly homogenize the sister copy genes, which would thus appear as to have more recent common ancestry in the phylogenetic tree (Fig. S1, S2). Under the repeated gene duplication model, the ancestral sequence in the evolutionary time window that predates the different duplication events should be the same for the *fem* and *csd*/*fem1* genes because at this time point, only a single copy of the gene existed (Fig. 2a).

We generated separate phylogenetic trees using the amino acid sequence of the Fem and Fem1/Csd proteins, which allowed us to trace the putative separate evolutionary history of these sister copy genes. We inferred the ancestral amino acid sequences of the MRCAs of bees and of ants (Fig. 2, S2) using the maximum likelihood method [22]. These nodes had high statistical support and represented evolutionary time windows before the putative repeated gene duplication events. For the large evolutionary distances between ants and bees, we analyzed amino acid changes instead of synonymous substitutions, which were saturated, at least for the less degenerate sites. We identified changes in the Fem protein of the MRCA amino acid sequence of bees and ants by comparing the MRCA sequences of bees and of ants. We found 7 changes in the MRCA sequence of bees and 9 in the MRCA sequence of ants. In ants, we found the same 9 amino acid changes in the sister copy sequence of the paralogous Fem1 protein (Fig. 2b, Fig. S3). In bees, we found the same 7 amino acid changes in the MRCA sequence of the paralogous Csd/Fem1 protein (Fig. 2c, Fig. S4). These informative changes in the ant and bee sequences are found in different parts of the protein, suggesting that we obtained information that covered the entire protein. Our study found no amino acid changes that were confined to only one of the sister copies, which would indicate a deeper ancestry of the gene duplication that predated the MRCA of bees and of ants. Therefore, the sequences harbor no information that can provide evidence for a separate history of the *fem* and *csd*/*fem1* genes that predates those of the ant and bee lineages, or for a single gene duplication event in Hymenoptera.

### No evidence of concerted evolution is found in bumblebee sequences

**Figure 3:**
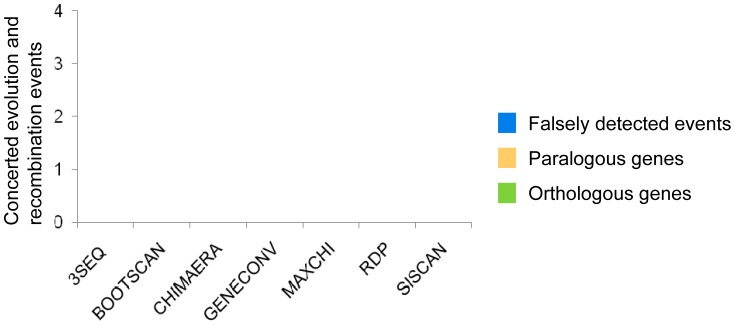
Number of gene conversion and recombination events in *B. terrestris* and *B. impatiens* sequences. Tracts of putatively recombined sequence were detected by the 7 methods as shown on the *x*-axis and using RDP 3.44 software program. The analysis was run on a single alignment of the *fem* and *fem1* sequences of the *B. terrestris*, *B. impatiens* and *A. mellifera fem* sequence. Gene conversion events refer to DNA transfers between the paralogous genes *fem* and *fem*1. Recombination events indicate transfer events between sequences of the same gene. Falsely detected events (FDE) refer to biologically implausible events (see Materials and Methods).

We studied whether we can detect sequence tracts that would indicate a transfer of sequences between paralogous genes (concerted evolution) in the bumblebee lineage. The phylogenetic clustering of the *fem* and *fem1* sequences in the bumblebee lineage (Fig. S1) suggests that concerted evolutionary events should also occur in the bumblebee lineage. We used seven methods (RDP, GENECONV, BootScan, MaxChi, Chimaera, SiScan and 3Seq) designed to detect tracts of recombination events in the nucleotide sequences, which are included in the RDP 3.44 software package [23]. We applied this method to a single sequence alignment, which included the *fem* and *fem*1 sequences of the two *Bombus* species and the *fem* sequence from *A. mellifera* as an outgroup reference. We classified the detected transfer events as either (i) concerted evolution events if they occurred between paralogous genes, (ii) recombination events if they occurred between the same gene, and (iii) falsely discovered events (FDE) if these events are biologically implausible. Such biologically implausible events are events in which sequence transfer between orthologous genes should give rise to a paralogous recombinant sequence or are events in which the sequence of the outgroup reference species is involved. The results of the analysis are shown in Figure 3. We found no sequence tracts that were transferred between paralogous genes, suggesting that concerted evolution played no role in the evolution of the paralogous genes of the *Bombus* lineage.

**Figure 4:**
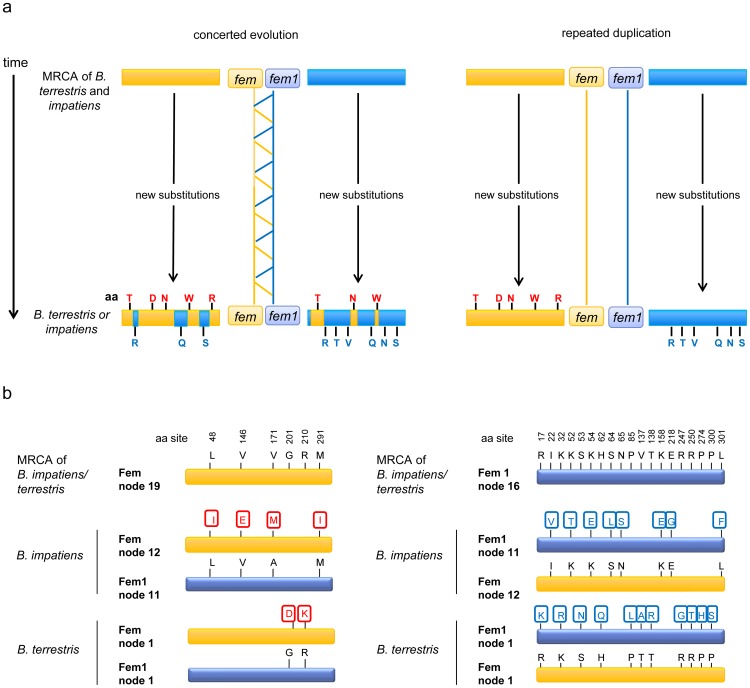
The evolutionary trajectory of *fem* gene substitutions in the evolutionary window that follows the putative gene duplication event in the *Bombus* lineage. (**a**) The expected evolutionary fate of *fem* substitutions in the paralogous genes *fem* and *fem1* under the models of concerted evolution and repeated gene duplication. (**b**) Deduced amino acid changes. The yellow box indicates the Fem protein, and the blue box indicates the Fem1 protein. Black letters above the boxes indicate the ancestral state of the amino acid residues found in the MRCA of *B. terrestris* and *B. impatiens*. Red letters in the red frame indicate the amino acid residues that evolved since the MRCA of *B. terrestris* and *B. impatiens* in the Fem protein. Blue letters in the blue frame indicate the amino acid residues that evolved since the MRCA of *B. terrestris* and *B. impatiens* in the Fem1 protein. Numbers before the boxes indicate the nodes (Fig. S2) used to infer the ancestral sequence information. aa denotes amino acid.

We next studied whether we can identify signatures of concerted evolution by confining our analysis to single amino acid substitutions. We tested whether some *fem* or *fem1* substitutions that newly evolved in each bumblebee species were transferred to its paralogous sister gene (Fig. 4a). Such shared evolved states between the two paralogous genes within each species would indicate a transfer of the corresponding nucleotide sequence by concerted evolution. We determined the ancestral sequences of the Fem and Fem1 protein of the MRCA of *Bombus terrestris* and *Bombus impatiens*, identified the evolutionary changes and studied whether these changes were also present in the paralogous sister copy (Fig. 4a). We found 4 newly evolved amino acid changes in the Fem protein of *B. impatiens* and 2 in that of *B. terrestris*. All of these newly evolved changes were not present in the sister Fem1 proteins (Fig. 4b, Fig. S5). We detected 8 newly evolved amino acid changes in the Fem1 protein of *B. impatiens* and 11 in *B. terrestris*, and these newly evolved changes were not present in the sister Fem protein (Fig. 4b, Fig. S6). Taken together, we found that all 12 newly evolved amino acid changes in *B. impatiens* and all 13 newly evolved amino acid changes in *B. terrestris* were absent in their corresponding sister copies. This survey covered different parts of the approximately 400 amino acid (aa)-long protein, providing evidence that concerted evolution events were absent in the *Bombus fem* and *fem1* sequences.

### No evidence of concerted evolution in sequences of the honeybee lineage

Next we applied the RDP, GENECONV, BootScan, MaxChi, Chimaera, SiScan and 3Seq methods to identify sequence tracts of concerted evolution between the paralogous *fem* and *csd* nucleotide sequences of the honeybee. We included the same coding nucleotide sequences as in a previous study [13], comprised of 36 *csd* and 1 *fem A. mellifera*, 16 *csd* and 1 *fem Apis cerana csd*, 19 *csd* and 1 *fem Apis dorsata* sequence and a *fem B. terrestris* sequence as an outgroup reference.

**Figure 5:**
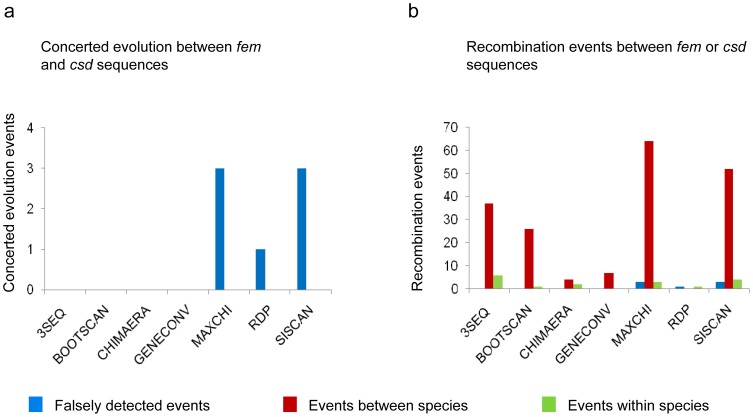
Events of gene conversion and recombination in the *fem* and *csd* sequences of *A. mellifera*, *A. dorsata* and *A. cerana*. Tracts of putatively recombined sequences were detected by seven different methods as indicated on the *x*-axis using the RDP 3.44 software program. The analysis was run on a single alignment of 71 *csd* and 4 *fem* sequences. (a) The number of concerted evolution events that refer to the DNA transfer between paralogous genes *fem* and *csd*. (b) The number of recombination events that identify events between sequences of the same gene. Falsely detected events (FDE) in (a) and (b) refer to biologically implausible events. The outgroup reference sequence, *B. terrestris fem*, was never involved in one of the detected events.

In this study, we applied the methods to a single sequence alignment that included all *fem* and *csd* sequences. In the previous study from Privman et al., 100 alignments were used, each consisting of the *fem* sequences and a randomly chosen *csd* allele sequence from each *Apis* species and a *fem* sequence from *B. terrestris*. The rationale behind our altered experimental design was that the detection methods used in the RDP 3.44 software program are designed for large datasets, to identify the recombinant sequence and the two sequences from which the recombinant sequence was derived [23]. We removed a sequence (GenBank accession #: AY352276) from the analysis because it was a chimeric sequence of the *csd* and *fem* gene. This sequence resulted from a misassembly of cDNA sequences derived from the *fem* and *csd* gene at a point in time when we had no knowledge about a second gene in the genome and the nature of allelic variation [3]. This sequence entry has now been removed from GenBank. We also updated the sequence (GenBank accession #: AY350616), which is a *fem* and not a *csd* sequence. The results of this sequence analysis are shown in (Fig. 5).

We found no tracts of gene conversion (Fig. 5a), suggesting that a DNA transfer between the *fem* and *csd* gene did not occur in the honeybee lineage. We observed 3 events in which recombination between the *Apis csd* ortholog sequences gave rise to a paralogous *Apis fem* sequence, suggesting falsely detected events (FDE) that were identified by the program. We confirmed this falsely detection by demonstrating that the putatively transferred fragment is indeed *fem* derived which we showed by the clustering into the *fem* gene cluster in the phylogenetic tree analysis. However, we detected multiple recombination events between the *csd* sequences (alleles) derived from the same and different *Apis* species (Fig. 5b). This suggests that recombination is a regular process between alleles of the *csd* gene, consistent with previous reports [7], [21].

We next evaluated how the number of sequences affects the false detection of events. We generated 20 sequence alignments, each consisting of two randomly chosen *csd* sequences from a single *Apis* species and one *fem* sequence from *B. terrestris*, which served as the outgroup reference sequence for the alignment above. For these alignments, the GENECONV method detected 8, the MaxChi method detected 12, and the Chimaera method detected 9 tracts of sequences in which DNA was putatively transferred between the *csd* sequences of the *Apis* and *fem* gene of the *B. terrestris* sequences (Fig. S7). However, these events are biologically implausible, because the nucleotide differences we observe today in the *csd* alleles evolved after the split into different *Apis* species [7]. Hence, the *Bombus* sequence cannot have contributed through concerted evolutionary events to the *csd* polymorphism. We confirmed this falsely detection in a sample of detected events by demonstrating that the putatively transferred fragment is not derived from the *Bombus* sequence, which we showed by the clustering into the *csd* gene cluster in the phylogenetic tree analysis. We detected no such transfer between the *Apis csd* alleles and the *B. terrestris fem* sequences if all sequences are included in a single alignment (Fig. 5) suggesting that having fewer sequences in an alignment increases the rate of falsely detecting DNA transfer between paralogs.

We did not perform single amino acid substitution analysis in the honeybee as we did for *Bombus* because we have not robustly identified enough newly evolved sites.

### d_N_/d_S_ ratio differences between paralogous genes suggest a directional DNA transfer process

We consistently observed that the *d_N_/d_S_* ratios (nonsynonymous (*d_N_*) to synonymous (*d_S_*) per site substitutions) of the *fem1*/*csd* sequence pairs were higher compared to those of the *fem* sequence pairs ((Table 1); χ^2^-test,*P*<0.05 for all comparisons), suggesting that selection operates differently on the *fem* and the *fem1/csd* genes. The ratio of the differences is most pronounced for the *Apis* sequences (*d_N_/d_Sfem_* = 0.1–0.2 versus *d_N_/d_Scsd_* = 0.8–1), consistent with previous findings [4], [8], and for the bumblebee sequences (*d_N_/d_Sfem_* = 0.16 versus *d_N_/d_Scsd_* = 0.56). The difference in the *d_N_/d_S_* ratios is less pronounced in the ants.

**Table 1:** The *d_N_* and *d_S_* values and ratios for the interspecies comparisons of the *fem* and *fem1/csd* genes.

			*fem* gene			*fem1/csd* gene			*d_N_*/*d_Sfem_*
Clade	Species		dN (SE)	dS (SE)	dN/dS	dN (SE)	dS (SE)	dN/dS	<*d_N_*/*d_Sfem_*_ 1/*csd*_ *
*Apis*	*Ador*	*Amel*	0.02 (0.004)	0.09 (0.02)	0.22	0.13 (0.01)	0.13 (0.02)	1.00	*P*<0.0001
	*Amel*	*Acer*	0.01 (0.003)	0.08 (0.02)	0.13	0.12 (0.01)	0.15 (0.02)	0.8	*P*<0.0001
*Bombus*	*Bter*	*Bimp*	0.008 (0.003)	0.051 (0.013)	0.16	0.05 (0.007)	0.09 (0.02)	0.56	*P*<0.01
	*Cflo*	*Hsal*	0.26 (0.01)	0.51 (0.029)	0.51	0.35 (0.02)	0.5 (0.03)	0.7	*P*<0.01
	*Cflo*	*Pbar*	0.19 (0.01)	0.44 (0.03)	0.43	0.3 (0.01)	0.43 (0.03)	0.7	*P*<0.01
Ants	*Hsal*	*Pbar*	0.24 (0.01)	0.53 (0.03)	0.45	0.26 (0.01)	0.46 (0.03)	0.57	*P*<0.02
	*Acep*	*Pbar*	0.11 (0.01)	0.37 (0.03)	0.30	0.16 (0.01)	0.41 (0.03)	0.39	*P*<0.05
	*Cflo*	*Acep*	0.21 (0.01)	0.46 (0.03)	0.46	0.32 (0.01)	0.49 (0.03)	0.65	*P*<0.01
	*Acep*	*Hsal*	0.23 (0.01)	0.5 (0.03)	0.46	0.28 (0.01)	0.44 (0.03)	0.64	P<0.01

Species names: *Amel*, *A. mellifera*; *Ador*, *A. dorsata*; *Acer*, *A. cerana*; *Bimp*, *B. impatiens*; *Bter*, *B. terrestris*; *Hsal*, *H. saltator*; *Pbar*, *P. barbatus*; *Acep*, *Atta cephalotes*; *Cflo*, *Camponotus floridanus*. SE: standard error.

*A one-tailed χ^2^ - test was conducted using the absolute number of synonymous and nonsynonymous differences.

We further evaluated how differences in the *d_N_/d_S_* ratios are compatible with the mutual transfer of DNA and concerted evolution. The mutual transfer between the paralogous genes would also transfer the differences in the *d_N_/d_S_* ratios between paralogous genes. We assume that the entire sequences are in equilibrium of homogenization through concerted evolution and divergence. This is consistent with the model that concerted evolution is a random mutational and ongoing process that occurs through hymenopteran phylogeny. At this equilibrium, the gene-wide *d_N_/d_S_* values are good approximations for DNA fragments that are, on average, transferred between paralogs.

First, we showed that there are not different rates at synonymous sites in the honeybee and the bumblebee clade (Tajima's relative rate test, P>0.05). This result suggests that the differences in the *d_N_/d_S_* ratios between the genes reflect substitution rate differences at nonsynonymous sites (*d_N_*).

DNA fragments transferred from the *fem1*/*csd* can only reach the lower *d_N_/d_S_* ratios in the *fem* gene as evolutionary time progresses if new mutations occur at the neutral synonymous sites along with purifying selection at the nonsynonymous sites. We approximated the mean number of neutral pairwise substitutions per site (*d_S_*) that is required to reach the lower *d_N_/d_S_* ratios. The *csd* sequences in honeybees show an average ratio of *d_N_/d_S csd_* = 0.9 (Table 1), suggesting that during the separation time of the two paralogous genes (in terms of *d_Scsd/fem_* = 0.18, Table S1), approximately *d_N csd_* = 0.16 pairwise substitutions in the *csd* gene have accumulated. We next estimated *d_S_*, which has accumulated so that a transferred *csd* fragment (that has on average a ratio *d_N_/d_S csd_* = 0.9) can reach the observed *d_N_/d_S_* ratio of the *fem* gene (in which *d_N_/d_S fem_* = 0.17). We assume the most conservative model, in which all newly arising nonsynonymous mutations were removed by purifying selection and only new synonymous mutations became fixed. A *csd* fragment can only adjust for the *fem*'s *d_N_/d_S_* ratio if *d_S x_* = 0.77 additional synonymous substitutions have accumulated. This result suggests that a transferred *csd* DNA requires on average *d_S_* = 0.77 synonymous substitutions to observe the low *d_N_/d_S_* ratio of the *fem* gene.

Similarly, we approximated *d_S x_* for bumblebee sequences. *fem1*-derived sequences in the *fem* sequence (*d_S fem1/fem_* = 0.22, *d_N_/d_S fem1_* = 0.56, *d_N fem1_* = 0.12, *d_N_/d_S fem_* = 0.16) would require *d_S x_* = 0.53 pairwise synonymous differences to accumulate in order for the sequence to reach the same *d_N_/d_S fem_* ratio.

*d_S x_* = 0.77 in honeybees and *d_S x_* = 0.53 in bumblebees, as required for the transferred *csd* DNA to reach the observed *d_N_/d_S_* ratios of the *fem* gene are largely exceeding *d_S_* = 0.39 that have accumulated between the bumblebee and honeybee species. This suggests that these transfers should predate the MRCA of bees, which is inconsistent with our previous result that there would be an absence of such transfers in this evolutionary window (Fig. 2). In addition, *d_S x_* largely exceeds the divergence between paralogous genes in the bumblebee (*d_S fem1/fem_* = 0.22) and the honeybee (*d_S fem/csd_* = 0.18), suggesting that the divergence between paralogs is too low to be compatible with such transfers and ratios. The results of this simple transfer model imply that the transfer processes cannot be bidirectional between paralogous genes, as predicted under a model of concerted evolution in which sequences are mutual exchanged and can become fixed through positive selection and genetic drift. Only a directional transfer from *fem* to *csd/fem1* would be compatible with data suggesting that relaxed or positive selection could substantially increase the *d_N_/d_S_* ratio. This directional transfer process is consistent with the gene duplication model, in which a new gene copy becomes neofunctionalized [4].

### Additional sequence copies are repeatedly found at other genomic loci in bees and ants

**Figure 6:**
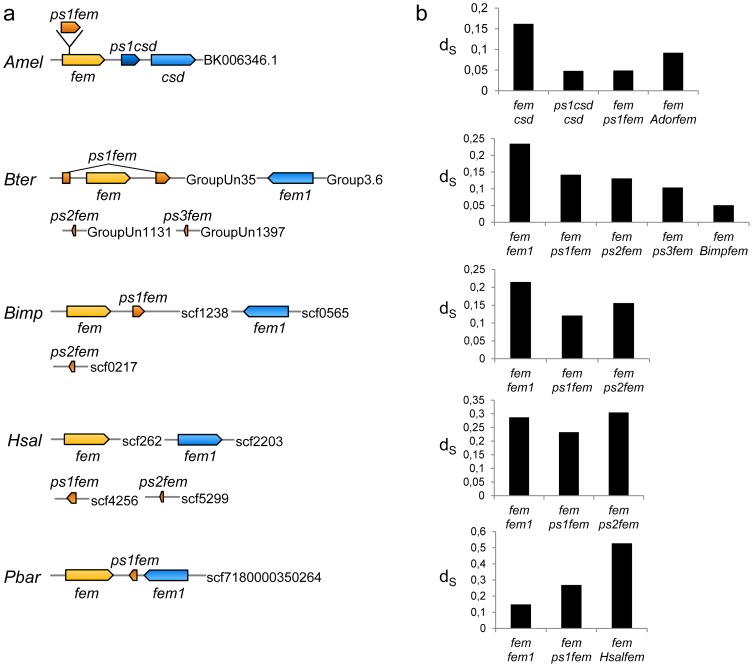
Pseudogenes (*ps*) of the *fem* and *csd* genes in the ant, bumblebee and western honeybee genomes. (**a**) The orientation and location of the pseudogenes (*psfem*, *pscsd*). Boxes denote the genes or pseudogenes. The box length of pseudogenes indicates the relative degree of homology to the coding nucleotide sequences of the *fem* or *csd* genes. The phylogenetic relationship assignments are based on the lowest *d_S_* estimates or the ancestral state. Numbers behind the bars indicate the genomic scaffold, linkage group or the GenBank accession number. (**b**) Evolutionary distance between duplicated *fem* and *fem1/csd* gene copies are presented in terms of pairwise synonymous divergence per synonymous site (*d_S_*). Abbreviations*: Amel*, *A. mellifera*; *Bimp*, *B. impatiens*; *Bter*, *B. terrestris*; *Hsal*, *H. saltator*; *Pbar*, *P. barbatus.*

To find further evidence for the repeated duplication model, we searched for other gene duplication tracts of the *fem* and *fem1/csd* genes. Using BlastN searches, we found genomic sequences with a high similarity to the coding nucleotide sequences of the *fem* or *csd* genes in the western honeybee, two bumblebee species and two ant species at other genomic loci (Fig. 6a). These duplicate genes exist as inactive genes with no complete open reading frame (ORF), suggesting that they are pseudogenes (*ps*). For the *ps1 csd* gene of *A. mellifera*, we confirmed by RT-PCR that this gene is transcriptionally inactive. We estimated the synonymous pairwise divergence (*d_S_*) of the pseudogenes and of the *fem* or *fem1/csd* genes within each species (Fig. 6b), which we related to the MRCA events by providing the *fem d_S_* values of different species. For *A. mellifera*, we found two pseudogenes, one derived from the *csd* gene and one derived from the *fem* gene (Fig. 6). The latter of these gave rise to a new female intron sequence in the *fem* gene [5]. The *d_S_* divergence between pseudogenes and the *fem* or *csd* gene is smaller compared to the *d_S_* between the *csd* and *fem* and the *d_S_* between the *fem* of *A. mellifera* and *A. dorsata* (Fig. 6b), suggesting that both sequences were duplicated recently in the *A. mellifera* lineage. The *d_S_* divergence in the bumblebee genomes suggests that the origin of these duplications predates the split between the *B. terrestris* and *B. impatiens* species (Fig. 6b) but that it originated after the split of the current functional gene copies of *fem* and *fem1*. In the ant *Harpegnathos saltator*, we observed one pseudogene that originated after and one that arose during the split between the functional *fem* and *fem1/csd* gene copies. However, in the ant *Pogonomyrmex barbatus*, we also found a pseudogene with a much deeper ancestry (*Pbarps1 fem*; *d_S_* = 0.27) than the functional gene pairs. Our results demonstrate that other gene duplication events occurred throughout the phylogeny and even within the *A. mellifera* lineage.

## Discussion

Our study presents several lines of evidence that support the repeated gene duplication model, but reject the concerted evolution model in which the low divergence of paralogs resulted from homogenization. We studied *fem* and *csd*/*fem1* paralogous genes in several bee and ant species, representing 120 million years of evolution [17]–[20]. We first showed that there were no unique changes in the Fem or Csd/Fem1 proteins at a point in time that would indicate a separate history of the two gene copies, predating the MRCAs of bees and of ants. We detected no sequence tracts that would indicate a DNA transfer between the paralogs in two bumblebee and three honeybee species by using different methods. We also identified other tracts of duplicated copies of the *fem* and *fem1*/*csd* gene at other genomic loci in different ant and bee species, suggesting that repeated gene duplication is a frequent process in the evolution of these genes. Finally, we showed that the major differences in the *d_N_/d_S_* ratio between the *fem* and *fem1*/*csd* genes in bees exclude a mutual transfer of sequences but suggest a directional transfer from *fem* to the *fem1*/*csd* gene, which is consistent with gene duplication and a neofunctionalization model [4] and not with a mutual exchange of sequences under concerted evolution. We conclude from these results that the *fem1*/*csd* genes repeatedly originated through gene duplication in the bumblebee, honeybee and ant lineages. Concerted evolution played no detectable role in the evolution of these genes, suggesting that the phylogenetic relationship of the paralogs is represented by a gene tree based on neutral synonymous sites (Fig. S1).

Our finding is consistent with frequency estimates of gene duplication and concerted evolution events. Previous studies estimated that a gene will, on average, duplicate every 100 million years [15], [16], which is consistent with our finding of repeated gene duplications of the *fem* gene in the phylogeny of ants and bees which split approximately 120 million years ago [17]–[20]. Another study showed that gene conversion is a rare event, detectable in only 2% of duplicated genes, and that this process requires physical distances smaller than 9 kb [14]. Contrary to the latter requirement, *fem* and *csd* gene are separated by more than 12 kb in the honeybee (*A. mellifera*). Studies of genes that have multiple copies in the genome have demonstrated that new copies constantly originate by gene duplication [24]–[35]. Some of the duplicated copies are maintained in the genome for an extended period of time, while other copies were deleted or became nonfunctional through the accumulation of deleterious mutations.

Previous studies [11], [13] proposed that concerted evolution produced the low divergence of the *fem* and *fem1*/*csd* genes. The authors suggested that (i) alternative tree topologies of the nucleotide sequences [13] and (ii) putative gene conversion tracts [11], [13] are evidence for concerted evolution. Schmieder et al. identified gene conversion tracts in the genomic sequences of the paralogs. Our results showed that *de novo* duplications of the *fem* and *fem1*/*csd* genes generated such tracts. Privman et al. counted more than 100 recombination events in the honeybee *fem* and *csd* sequences, which they take as evidence for concerted evolution. In our reanalysis of the same sequences using the same methods (Fig. 5), we distinguished whether the transfer of DNA occurred between paralogous genes (concerted evolution) or between alleles of the same (orthologous) gene (recombination events). We found no tracts of gene conversion events between the *fem* and *csd* sequences (Fig. 5a), suggesting the absence of concerted evolution in honeybees. However, we found repeated transfers between alleles of the same (*csd*) gene (Fig. 5b), a finding which has been repeatedly reported [7], [21].

In our reanalysis, we used the entire 75-sequence data set in a single alignment, in contrast to the Privman et al. study, which used 100 alignments of 7 *fem* and *csd* sequences with different sets of *csd* alleles chosen from each species. We inspected some of the results using the Privman et al. alignments and repeatedly found tracts that suggested recombination events between the *csd* alleles. We also demonstrated in this study that, as in the work of Privman et al., small alignments of only a few sequences (Fig. S7) can generate an increase of the number of falsely detected events.

Privman et al. also proposed that the differences in the phylogenetic relationships of recombinant and non-recombinant regions provide further evidence for concerted evolution. Because these falsely detected “recombinant” sequence tracts are sequences from the same gene (*csd*) (see Fig. 5) these inconsistencies in the phylogenetic relationships are no further evidence of concerted evolution events. These “inconsistencies” have been previously reported for *csd* alleles. The combined forces of meiotic recombination and balancing selection generate a heterogeneity of divergence across the *csd* gene [6], [7], [21]. Recombination redistributes a small subset of variants of the 5′ region with multiple, highly diverged 3′ variants, which generates inconsistencies in the resulting phylogenetic relationships as previously shown [7], [21], [36].

Privman et al. found *fem*-specific substitutions for *csd* allele AY352276. However, this sequence is actually a chimeric sequence that was generated by the misassembly of the *fem* and *csd* cDNA sequences. This sequence was generated at a point in time when we searched for a third *csd* allele and had no knowledge of a second gene or the nature of the allelic diversity [3]. This sequence entry has been removed from GenBank. Privman et al. also suggested that alternative tree topologies of *fem* and *fem1* sequences in the ants indicate gene conversion events [13]. We argue that the divergence of these sequences is too high (*d_S_* = 0.5 for most species pairs) to exclude the possibility that ambiguous trees (and split phylogenetic networks) resulted from homoplasic (convergent) substitutions in the sequences.

Our results imply that the *fem1* and *csd* genes in the ant, bumblebee and honeybee species are not orthologs because they originated independently through gene duplications (Fig. S1). The *csd* gene originated in the honeybee lineage [4], [5]. Hence, complementary sex determination in bumblebee and ant species [1], [2] is regulated by other genes and not by the orthologs of the *csd* gene. Consistent with this conclusion, the *csd* alleles of honeybees share a hypervariable region of asparagine- and tyrosine-enriched repeats [7] that are consistently absent in the *fem1* genes of ant and bumblebee species.

Our results show that a new gene for complementary sex determination originated in honeybees [4], while the phylogenetic distribution of complementary sex determination indicates a deep ancestry in Hymenopteran insects [1], [2]. One explanation for the replacement of a complementary sex determination gene is that ancestral complementary sex determiner genes degenerate over evolutionary time [4], [10]. This is because meiotic recombination is suppressed at the sex determiner gene locus [21], allowing more deleterious mutations to accumulate over time [37]–[39]. This process could generate an adaptive advantage for evolving new sex determination genes that would eventually replace the older, malfunctioning, complementary sex determination gene. Such a degeneration process has been proposed for sex chromosomal systems [40]–[45] and may also explain the rapid evolution of the complementary sex determination system.

Characterizing the gene functions of the *fem1* genes and the molecular basis of complementary sex determination in ants and bumblebees would provide interesting insights into the evolution of this sex determination system. Our results imply that the conserved phenotype (complementary sex determination) is only loosely evolutionary associated with the controlling molecular process. These findings highlight the limits of comparative genomics and emphasize the requirement to study gene functions in different species and major hymenopteran lineages.

## Materials and Methods

The *fem* paralogous sequences defined in this study as *fem1* (in ants and bumblebees) or *csd* (in honeybees) were taken from Schmieder et al. [11] and were provided by Schmieder, S. and Poirie, M. The genomic locus of the *fem*/*fem1* gene of *Solenopsis invicta* was not accessible in public libraries. The honeybee *fem* and *csd* sequences that we used to detect tracts of DNA transfers were kindly provided by Privman, E. The coding sequences were aligned based on the deduced amino acids, assuming a standard genetic code table. We used either the Clustal or the Muscle program that was implemented in the MEGA5 program suite [46] to align the coding nucleotide sequences according to the deduced amino acid sequences. The alignments were edited manually. Nucleotide and amino acid substitution analyses were conducted using the MEGA5 program. Maximum likelihood fits of the substitution models with the lowest Bayesian information criterion (BIC) score were used to choose a substitution model for amino acids and nucleotides when possible. Pairwise gaps in the alignment were deleted.

### Ancestral sequences

Ancestral amino acid sequences were inferred using the maximum likelihood method [22] under the Jones-Taylor-Thornton (JTT) matrix-based model [47]. The rates between sites were treated as a gamma distribution. The ancestral sequences were inferred from separate *fem* and *fem1*/*csd* sequence alignments, and Figure S2 shows the gene tree used. All informative changes used had a probability *P*>0.5. We had to exclude some sites where the homology between sites of the Fem protein and the Csd/Fem1 alignment were ambiguous. In three cases of the bumblebee analysis, we changed the ancestral state we obtained from the MEGA analysis, because it contradicted the parsimony evolution of the sequence in the Fem and Fem1 tree (Fig. S5 and Fig. S6).

### Evolutionary divergence between sequences

The *d_N_/d_S_* ratios of the interspecies comparisons between the ants, bumblebees and honeybees were inferred from sequence alignments that included either the bee or ant sequences, which greatly improved the number of identical positions in the alignments. The evolutionary distances between the ants and bees suggested that the less degenerated synonymous sites were saturated, making the *d_S_* estimates between the bee and ant sequences less reliable. Analyses were conducted using the Nei-Gojobori model to estimate either the nonsynonymous and synonymous substitutions per site or the absolute numbers. A χ^2^-square test was used to test the ratio differences in terms of absolute numbers. We tested the equality of the evolutionary rate at the most degenerate third codon position (which is presumably largely synonymous) by using Tajima's relative rate [48], which we performed using MEGA5 software [46]. We tested equality among the *Apis csd* and *fem* sequences by using the *Bombus fem* sequence as the outgroup. Similarly, we tested equality among *Bombus fem1* and *fem* sequences by using the *Apis fem* sequence as an outgroup.

### RT-PCR analysis

We performed RT-PCR experiments on embryonic and larval RNA [5] to identify possible splice products of mRNA in regions of the identified pseudogenes. PCR fragments were sequenced and compared with the genomic data.

### Phylogenetic relationships

The phylogenetic relationship of the *fem* and *fem1/csd* sequences was determined based on presumably neutral synonymous differences by excluding the *tra* sequences of *Ceratitis capitata* and *Nasonia vitripennis* (because *d_S_* could not be estimated for these species) using the neighbor-joining method. The confidence probability (multiplied by 100) that the interior branch length is greater than 0 was estimated using the bootstrap test with 10,000 replicates. The evolutionary distances were computed using the Pamilo-Bianchi-Li method and are displayed in the units of the number of synonymous substitutions per synonymous site. For each sequence pair, all ambiguous positions were removed.

### Sequence tracts of DNA transfers

Tracts of DNA transfers in the honeybee *fem* and *csd* sequences were identified using the RDP 3.44 software program [23]. We used the following tests: RDP [49] with internal and external references, GENECONV [50], BootScan [51], MaxChi [52], Chimaera [53], SiScan [54] and 3Seq [55]. The methods implemented in the RDP 3.44 software program relied on the identification of recombinant sequences and the parental sequences from which these recombinant sequences were derived, which is facilitated by having a large set of sequences [23]. We thus generated a single alignment. Our alignment included 36 *A. mellifera*, 16 *A. cerana* and 19 *A. dorsata csd* coding nucleotide sequences, the single f*em* nucleotide sequence from each *Apis* species, and the *fem* nucleotide sequence of *B. terrestris* as an outgroup reference sequence. We used the same program settings as described in a previous study [13].We removed sequence GenBank accession #: AY352276 from the analysis, as this is a chimeric sequence of the *fem* and the *csd* gene (the entry has now been deleted). We also updated the sequence (GenBank accession #: AY350616) because it is not a *csd* but a *fem*-derived sequence. To evaluate the effect of the number of sequences in the alignment, we generated 20 sequence alignments consisting of three sequences. Each alignment included two randomly chosen *csd* sequences from a single *Apis* species as well as the *fem* sequence from *B. terrestris*. We classified the detected transfer events as (i) concerted evolution events if they occurred between paralogous genes, (ii) recombination events if they occurred between the same gene and (iii) falsely discovered events (FDE) if they were biologically implausible. Such biologically implausible events are events in which the sequence transfer between orthologous genes should give rise to a paralogous recombinant sequence or events in which the sequence of the outgroup reference species was involved. We confirmed falsely detection by inferring the clustering of the sequence tracts into the gene cluster in phylogenetic tree analyses.

## Supporting Information

Figure S1 **Gene tree of the** ***fem*** **and** ***fem1/csd*** **sister copies in ants and bees, which were inferred from synonymous differences.** The evolutionary history was inferred using the neighbor-joining method. The confidence probability (multiplied by 100) that the interior branch length is greater than 0 was estimated using the bootstrap test (10000 replicates are shown next to the branches). The tree is drawn to scale, with branch lengths in the same units as those of the evolutionary distances used to infer the phylogenetic tree. The evolutionary distances were computed using the Pamilo-Bianchi-Li method [1] and are in the units of the number of synonymous substitutions per synonymous site. All ambiguous positions were removed for each sequence pair. There were a total of 575 positions in the final dataset. Evolutionary analyses were conducted in MEGA 5 [2]. The sequences of *Nasonia* and *Ceratitis* were excludeto estimate *d_S_*. Abbreviations: *Acep*, *Atta cephalotes*; *Acer, Apis cerana*; *Aech*, *Acromyrmex echinatior*; *Ador, Apis dorsata*; *Amel*, *Apis mellifera*; *Bimp, Bombus impatiens*; *Bter*, *Bombus terrestris*; *Cflo*, *Camponotus floridanus*; *Hsal*, *Harpegnathos saltator*; *Mcom, Melipona compressipes*; *Pbar*, *Pogonomyrmex barbatus*; *Sinv, Solenopsis invicta*.
(DOCX)

Figure S2 **The initial tree of the Fem (a) and Fem1/Csd (b) proteins that were used to infer the ancestral amino acid sequences.** The evolutionary history was inferred by using the maximum likelihood method based on the JFF model [1]. Numbers in the tree assign the different nodes for which the ancestral sequence were obtained. The tree is drawn to scale, with branch lengths measured in the number of substitutions per site. Abbreviations: *Acep*, *Atta cephalotes*; *Acer, Apis cerana*; *Aech*, *Acromyrmex echinatior*; *Ador, Apis dorsata*; *Aflo*, *Apis florea*; *Amel*, *Apis mellifera*; *Bimp, Bombus impatiens*; *Bter*, *Bombus terrestris*; *Ccap, Ceratitis capitata*; *Cflo*, *Camponotus floridanus; Hsal*, *Harpegnathos saltator*; *Mcom, Melipona compressipes*; *Nvit, Nasonia vitripennis*; *Pbar*, *Pogonomyrmex barbatus*; *Sinv, Solenopsis invicta*.
(DOCX)

Figure S3 **The informative substitutions found in the ant lineage that were used in** **Figure 2** **.** The identity of the different species and nodes of the Fem and Fem1 protein tree is shown. Site number (#) indicates the positions in the Fem and in the Csd/Fem1 protein sequence alignment.
(DOCX)

Figure S4 **The informative substitutions found in the bee lineage that were used in** **Figure 2** **.** The identity of the different species and nodes of the Fem and Csd/Fem1 protein tree is shown. Site number (#) indicates the positions in the Fem and in the Csd/Fem1 protein sequence alignment.
(DOCX)

Figure S5 **The informative substitutions found in the** ***Bombus*** **lineage (Fem tree) that were used in** **Figure 3** **.** The identity of the different species and nodes of the Fem and Fem1 protein tree is shown. Site number (#) indicates the position in the alignment of the Fem and of the Csd/Fem1 protein sequences.
(DOCX)

Figure S6 **The informative substitutions found in the** ***Bombus*** **lineage (Fem1 tree) that were used in** **Figure 3** **.** The identity of the different species and nodes of the Fem and Fem1 protein tree is shown. Site number (#) indicate the position in the alignment of the Fem and of the Csd/Fem1 protein sequences.
(DOCX)

Figure S7 **The number of falsely detected events (FDE) using the methods as indicated on the X axis.** These programs were implemented in the RDP 3.44 software program. The methods were run on each of the 20 alignments which consisted of two randomly chosen *csd* sequences from a single *Apis* species and one *fem* sequence from *B. terrestris*. These events are falsely detected as this transfer involve the outgroup *Bombus* sequence and the polymorphism between csd alleles which newly evolved in the different *Apis* species.
(DOCX)

Table S1 The *d_S i_* values for the paralogous gene pairs *fem* and *csd*/*fem*1 within each species (*Ador, Apis dorsata*; *Amel*, *Apis mellifera*; *Bimp, Bombus impatiens*; *Bter*, *Bombus terrestris*).
(DOCX)
