# Supplementary material for: Independent Evolutionary Origin of fem Paralogous Genes and Complementary Sex Determination in Hymenopteran Insects
Source: PLoS One. 2014 Apr 17;9(4):e91883. doi: 10.1371/journal.pone.0091883 (PMC3990544; doi:10.1371/journal.pone.0091883)
Supplement: Figure S1 — Gene tree of the fem and fem1/csd sister copies in ants and bees, which were inferred from synonymous differences. The evolutionary history was inferred using the neighbor-joining method. The confidence probability (multiplied by 100) that the interior branch length is greater than 0 was estimated using the bootstrap test (10000 replicates are shown next to the branches). The tree is drawn to scale, with branch lengths in the same units as those of the evolutionary distances used to infer the phylogenetic tree. The evolutionary distances were computed using the Pamilo-Bianchi-Li method [1] and are in the units of the number of synonymous substitutions per synonymous site. All ambiguous positions were removed for each sequence pair. There were a total of 575 positions in the final dataset. Evolutionary analyses were conducted in MEGA 5 [2]. The sequences of Nasonia and Ceratitis were excludeto estimate dS. Abbreviations: Acep, Atta cephalotes; Acer, Apis cerana; Aech, Acromyrmex echinatior; Ador, Apis dorsata; Amel, Apis mellifera; Bimp, Bombus impatiens; Bter, Bombus terrestris; Cflo, Camponotus floridanus; Hsal, Harpegnathos saltator; Mcom, Melipona compressipes; Pbar, Pogonomyrmex barbatus; Sinv, Solenopsis invicta. (DOCX) [file pone.0091883.s001.docx]

***Acer* Csd**

***Ador* Csd**

***Amel* Csd**

***Ador*Fem**

***Acer*Fem**

***Amel*Fem**

***Mcom*Fem**

***Bter* Fem1**

***Bimp* Fem1**

***Bter* Fem**

***Bimp*Fem**

***Pbar* Fem1**

***Pbar*Fem**

***Cflo* Fem1**

***Cflo*Fem**

***Hsal* Fem1**

***Hsal*Fem**

***Sinv*Fem**

***Acep* Fem1**

***Acep*Fem**

***Aech*Fem**

93

94

78

94

90

77

70

50

93

75

93

92

95

18

53

77

86

0.1

**Figure S1**. Gene tree of the *fem* and *fem1/csd* sister copies in ants and bees, which were inferred from synonymous differences. The evolutionary history was inferred using the neighbor-joining method. The confidence probability (multiplied by 100) that the interior branch length is greater than 0 was estimated using the bootstrap test (10000 replicates are shown next to the branches). The tree is drawn to scale, with branch lengths in the same units as those of the evolutionary distances used to infer the phylogenetic tree. The evolutionary distances were computed using the Pamilo-Bianchi-Li method [[1](#_ENREF_1)] and are in the units of the number of synonymous substitutions per synonymous site. All ambiguous positions were removed for each sequence pair. There were a total of 575 positions in the final dataset. Evolutionary analyses were conducted in MEGA 5 [[2](#_ENREF_2)]. The sequences of *Nasonia* and *Ceratitis* were excludeto estimate *d_S._* Abbreviations: *Acep*, *Atta cephalotes*; *Acer, Apis cerana*; *Aech*, *Acromyrmex echinatior*; *Ador, Apis dorsata*; *Amel*, *Apis mellifera*; *Bimp, Bombus impatiens*; *Bter*, *Bombus terrestris*; *Cflo*, *Camponotus floridanus; Hsal*, *Harpegnathos saltator*; *Mcom, Melipona compressipes*; *Pbar*, *Pogonomyrmex barbatus*; *Sinv, Solenopsis invicta*.

1. Pamilo P, Bianchi NO (1993) Evolution of the Zfx and Zfy genes: rates and interdependence between the genes. MolBiolEvol 10: 271-281.

2. Tamura K, Peterson D, Peterson N, Stecher G, Nei M, et al. (2011) MEGA5: molecular evolutionary genetics analysis using maximum likelihood, evolutionary distance, and maximum parsimony methods. MolBiolEvol 28: 2731-2739.
