# Supplementary material for: Independent Evolutionary Origin of fem Paralogous Genes and Complementary Sex Determination in Hymenopteran Insects
Source: PLoS One. 2014 Apr 17;9(4):e91883. doi: 10.1371/journal.pone.0091883 (PMC3990544; doi:10.1371/journal.pone.0091883)
Supplement: Figure S2 — The initial tree of the Fem (a) and Fem1/Csd (b) proteins that were used to infer the ancestral amino acid sequences. The evolutionary history was inferred by using the maximum likelihood method based on the JFF model [1]. Numbers in the tree assign the different nodes for which the ancestral sequence were obtained. The tree is drawn to scale, with branch lengths measured in the number of substitutions per site. Abbreviations: Acep, Atta cephalotes; Acer, Apis cerana; Aech, Acromyrmex echinatior; Ador, Apis dorsata; Aflo, Apis florea; Amel, Apis mellifera; Bimp, Bombus impatiens; Bter, Bombus terrestris; Ccap, Ceratitis capitata; Cflo, Camponotus floridanus; Hsal, Harpegnathos saltator; Mcom, Melipona compressipes; Nvit, Nasonia vitripennis; Pbar, Pogonomyrmex barbatus; Sinv, Solenopsis invicta. (DOCX) [file pone.0091883.s002.docx]

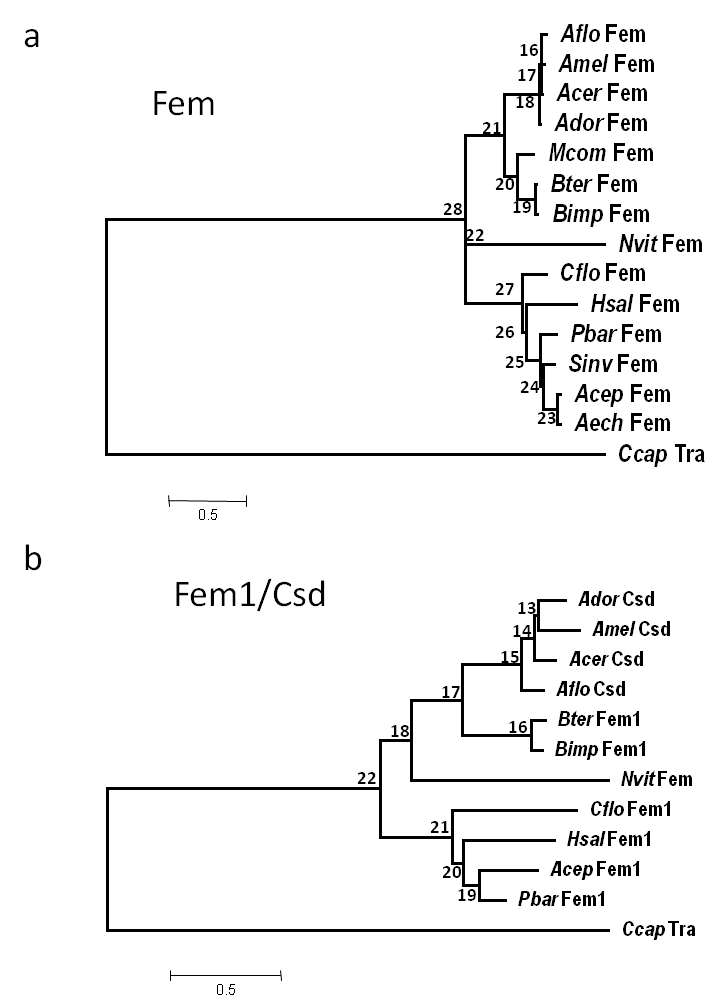


**Figure S2**. The initial tree of the Fem **(a)** and Fem1/Csd **(b)** proteins that were used to infer the ancestral amino acid sequences. The evolutionary history was inferred by using the maximum likelihood method based on the JFF model [[1](#_ENREF_1)]. Numbers in the tree assign the different nodes for which the ancestral sequence were obtained. The tree is drawn to scale, with branch lengths measured in the number of substitutions per site. Abbreviations: *Acep*, *Atta cephalotes*; *Acer, Apis cerana*; *Aech*, *Acromyrmex echinatior*; *Ador, Apis dorsata*; *Aflo*, *Apis florea*; *Amel*, *Apis mellifera*; *Bimp, Bombus impatiens*; *Bter*, *Bombus terrestris*; *Ccap, Ceratitis capitata*; *Cflo*, *Camponotus floridanus; Hsal*, *Harpegnathos saltator*; *Mcom, Melipona compressipes*; *Nvit, Nasonia vitripennis; Pbar*, *Pogonomyrmex barbatus*; *Sinv, Solenopsis invicta*.

1. Jones DT, Taylor WR, Thornton JM (1992) The rapid generation of mutation data matrices from protein sequences. ComputApplBiosci 8: 275-282.
