# Supplementary material for: Independent Evolutionary Origin of fem Paralogous Genes and Complementary Sex Determination in Hymenopteran Insects
Source: PLoS One. 2014 Apr 17;9(4):e91883. doi: 10.1371/journal.pone.0091883 (PMC3990544; doi:10.1371/journal.pone.0091883)
Supplement: Figure S3 — The informative substitutions found in the ant lineage that were used in Figure 2 . The identity of the different species and nodes of the Fem and Fem1 protein tree is shown. Site number (#) indicates the positions in the Fem and in the Csd/Fem1 protein sequence alignment. (DOCX) [file pone.0091883.s003.docx]

**Figure S3**. The informative substitutions found in the ant lineage that were used in Figure 2. The identity of the different species and nodes of the Fem and Fem1 protein tree is shown. Site number (#) indicates the positions in the Fem and in the Csd/Fem1 protein sequence alignment.

| **Node 27 – Fem** | **Node 28 – Fem** | **Node21 – Fem1** |
| --- | --- | --- |
| **Q** | **M** | **Q** |

| Position in Fem protein sequence alignment: # 24 | | | | | | | |
| --- | --- | --- | --- | --- | --- | --- | --- |
|  | *C.flo.* | *A.cep.* | *H.sal.* | *A.ech.* | *S.inv.* | *P.bar.* | *N.vit.* |
| Site in | **Q** | **Q** | **R** | **Q** | **Q** | **Q** | **M** |
| Fem tree | *B.ter.* | *B.imp.* | *A.cer.* | *A.dor.* | *A.flo.* | *A.mel.* | *M.com.* |
|  | **M** | **M** | **M** | **M** | **M** | **M** | **M** |

| Position in Csd/Fem1 protein sequence alignment: # 21 | | | | |
| --- | --- | --- | --- | --- |
| Site in Csd / | *C.flo.* | *A.cep.* | *H.sal.* | *P.bar.* |
| Fem1 tree | **Q** | **Q** | **I** | **Q** |

| **Node 27 – Fem** | **Node 28 – Fem** | **Node21 – Fem1** |
| --- | --- | --- |
| **R** | **I** | **R** |

| Position in Fem protein sequence alignment: # 25 | | | | | | | |
| --- | --- | --- | --- | --- | --- | --- | --- |
|  | *C.flo.* | *A.cep.* | *H.sal.* | *A.ech.* | *S.inv.* | *P.bar.* | *N.vit.* |
| Site in | **R** | **R** | **H** | **R** | **R** | **R** | **V** |
| Fem tree | *B.ter.* | *B.imp.* | *A.cer.* | *A.dor.* | *A.flo.* | *A.mel.* | *M.com.* |
|  | **I** | **I** | **I** | **I** | **I** | **I** | **I** |

| Position in Csd/Fem1 protein sequence alignment: # 22 | | | | |
| --- | --- | --- | --- | --- |
| Site in Csd / | *C.flo.* | *A.cep.* | *H.sal.* | *P.bar.* |
| Fem1 tree | **Q** | **R** | **R** | **R** |

| **Node 27 – Fem** | **Node 28 – Fem** | **Node21 – Fem1** |
| --- | --- | --- |
| **Q** | **E** | **Q** |

| Position in Fem protein sequence alignment: # 32 | | | | | | | |
| --- | --- | --- | --- | --- | --- | --- | --- |
|  | *C.flo.* | *A.cep.* | *H.sal.* | *A.ech.* | *S.inv.* | *P.bar.* | *N.vit.* |
| Site in | **Q** | **Q** | **Q** | **Q** | **Q** | **Q** | **E** |
| Fem tree | *B.ter.* | *B.imp.* | *A.cer.* | *A.dor.* | *A.flo.* | *A.mel.* | *M.com.* |
|  | **E** | **E** | **E** | **E** | **E** | **E** | **E** |

| Position in Csd/Fem1 protein sequence alignment: # 29 | | | | |
| --- | --- | --- | --- | --- |
| Site in Csd / | *C.flo.* | *A.cep.* | *H.sal.* | *P.bar.* |
| Fem1 tree | **Q** | **Q** | **Q** | **E** |

| **Node 27 – Fem** | **Node 28 – Fem** | **Node21 – Fem1** |
| --- | --- | --- |
| **T** | **S** | **T** |

| Position in Fem protein sequence alignment: # 81 | | | | | | | |
| --- | --- | --- | --- | --- | --- | --- | --- |
|  | *C.flo.* | *A.cep.* | *H.sal.* | *A.ech.* | *S.inv.* | *P.bar.* | *N.vit.* |
| Site in | **T** | **T** | **A** | **T** | **T** | **T** | **S** |
| Fem tree | *B.ter.* | *B.imp.* | *A.cer.* | *A.dor.* | *A.flo.* | *A.mel.* | *M.com.* |
|  | **S** | **S** | **S** | **S** | **S** | **S** | **S** |

| Position in Csd/Fem1 protein sequence alignment: # 77 | | | | |
| --- | --- | --- | --- | --- |
| Site in Csd / | *C.flo.* | *A.cep.* | *H.sal.* | *P.bar.* |
| Fem1 tree | **T** | **T** | **A** | **T** |

| **Node 27 – Fem** | **Node 28 – Fem** | **Node21 – Fem1** |
| --- | --- | --- |
| **T** | **A** | **T** |

| Position in Fem protein sequence alignment: # 96 | | | | | | | |
| --- | --- | --- | --- | --- | --- | --- | --- |
|  | *C.flo.* | *A.cep.* | *H.sal.* | *A.ech.* | *S.inv.* | *P.bar.* | *N.vit.* |
| Site in | **T** | **T** | **T** | **T** | **T** | **T** | **E** |
| Fem tree | *B.ter.* | *B.imp.* | *A.cer.* | *A.dor.* | *A.flo.* | *A.mel.* | *M.com.* |
|  | **A** | **A** | **A** | **A** | **A** | **A** | **I** |

| Position in Csd/Fem1 protein sequence alignment: # 91 | | | | |
| --- | --- | --- | --- | --- |
| Site in Csd / | *C.flo.* | *A.cep.* | *H.sal.* | *P.bar.* |
| Fem1 tree | **T** | **T** | **T** | **T** |

| **Node 27 – Fem** | **Node 28 – Fem** | **Node21 – Fem1** |
| --- | --- | --- |
| **T** | **A** | **T** |

| Position in Fem protein sequence alignment: # 155 | | | | | | | |
| --- | --- | --- | --- | --- | --- | --- | --- |
|  | *C.flo.* | *A.cep.* | *H.sal.* | *A.ech.* | *S.inv.* | *P.bar.* | *N.vit.* |
| Site in | **D** | **D** | **D** | **D** | **D** | **D** | **G** |
| Fem tree | *B.ter.* | *B.imp.* | *A.cer.* | *A.dor.* | *A.flo.* | *A.mel.* | *M.com.* |
|  | **N** | **N** | **N** | **N** | **N** | **N** | **N** |

| Position in Csd/Fem1 protein sequence alignment: # 150 | | | | |
| --- | --- | --- | --- | --- |
| Site in Csd / | *C.flo.* | *A.cep.* | *H.sal.* | *P.bar.* |
| Fem1 tree | **G** | **N** | **D** | **D** |

| **Node 27 – Fem** | **Node 28 – Fem** | **Node21 – Fem1** |
| --- | --- | --- |
| **S** | **L** | **S** |

| Position in Fem protein sequence alignment: # 159 | | | | | | | |
| --- | --- | --- | --- | --- | --- | --- | --- |
|  | *C.flo.* | *A.cep.* | *H.sal.* | *A.ech.* | *S.inv.* | *P.bar.* | *N.vit.* |
| Site in | **Q** | **S** | **S** | **S** | **S** | **S** | **L** |
| Fem tree | *B.ter.* | *B.imp.* | *A.cer.* | *A.dor.* | *A.flo.* | *A.mel.* | *M.com.* |
|  | **L** | **L** | **S** | **P** | **S** | **S** | **L** |

| Position in Csd/Fem1 protein sequence alignment: # 154 | | | | |
| --- | --- | --- | --- | --- |
| Site in Csd / | *C.flo.* | *A.cep.* | *H.sal.* | *P.bar.* |
| Fem1 tree | **S** | **S** | **S** | **S** |

| **Node 27 – Fem** | **Node 28 – Fem** | **Node21 – Fem1** |
| --- | --- | --- |
| **A** | **P** | **A** |

| Position in Fem protein sequence alignment: # 254 | | | | | | | |
| --- | --- | --- | --- | --- | --- | --- | --- |
|  | *C.flo.* | *A.cep.* | *H.sal.* | *A.ech.* | *S.inv.* | *P.bar.* | *N.vit.* |
| Site in | **A** | **A** | **A** | **T** | **A** | **T** | **P** |
| Fem tree | *B.ter.* | *B.imp.* | *A.cer.* | *A.dor.* | *A.flo.* | *A.mel.* | *M.com.* |
|  | **P** | **P** | **P** | **P** | **P** | **P** | **S** |

| Position in Csd/Fem1 protein sequence alignment: # 253 | | | | |
| --- | --- | --- | --- | --- |
| Site in Csd / | *C.flo.* | *A.cep.* | *H.sal.* | *P.bar.* |
| Fem1 tree | **A** | **A** | **M** | **T** |

| **Node 27 – Fem** | **Node 28 – Fem** | **Node21 – Fem1** |
| --- | --- | --- |
| **V** | **M** | **V** |

| Position in Fem protein sequence alignment: # 274 | | | | | | | |
| --- | --- | --- | --- | --- | --- | --- | --- |
|  | *C.flo.* | *A.cep.* | *H.sal.* | *A.ech.* | *S.inv.* | *P.bar.* | *N.vit.* |
| Site in | **V** | **V** | **M** | **V** | **V** | **V** | **L** |
| Fem tree | *B.ter.* | *B.imp.* | *A.cer.* | *A.dor.* | *A.flo.* | *A.mel.* | *M.com.* |
|  | **M** | **M** | **M** | **M** | **M** | **M** | **M** |

| Position in Csd/Fem1 protein sequence alignment: # 273 | | | | |
| --- | --- | --- | --- | --- |
| Site in Csd / | *C.flo.* | *A.cep.* | *H.sal.* | *P.bar.* |
| Fem1 tree | **V** | **V** | **M** | **V** |
