# Supplementary material for: Independent Evolutionary Origin of fem Paralogous Genes and Complementary Sex Determination in Hymenopteran Insects
Source: PLoS One. 2014 Apr 17;9(4):e91883. doi: 10.1371/journal.pone.0091883 (PMC3990544; doi:10.1371/journal.pone.0091883)
Supplement: Figure S4 — The informative substitutions found in the bee lineage that were used in Figure 2 . The identity of the different species and nodes of the Fem and Csd/Fem1 protein tree is shown. Site number (#) indicates the positions in the Fem and in the Csd/Fem1 protein sequence alignment. (DOCX) [file pone.0091883.s004.docx]

**Figure S4**. The informative substitutions found in the bee lineage that were used in Figure 2. The identity of the different species and nodes of the Fem and Csd/Fem1 protein tree is shown. Site number (#) indicates the positions in the Fem and in the Csd/Fem1 protein sequence alignment.

| **Node 21 – Fem** | **Node 22 – Fem** | **Node17 – Csd / Fem1** |
| --- | --- | --- |
| **T** | **S** | **T** |

| Position in Fem protein sequence alignment: # 74 | | | | | | | |
| --- | --- | --- | --- | --- | --- | --- | --- |
|  | *C.flo.* | *A.cep.* | *H.sal.* | *A.ech.* | *S.inv.* | *P.bar.* | *N.vit.* |
| Site in | **S** | **S** | **S** | **S** | **S** | **S** | **S** |
| Fem tree | *B.ter.* | *B.imp.* | *A.cer.* | *A.dor.* | *A.flo.* | *A.mel.* | *M.com.* |
|  | **T** | **T** | **T** | **T** | **T** | **T** | **T** |

| Position in Csd/Fem1 protein sequence alignment: # 69 | | | | | | |
| --- | --- | --- | --- | --- | --- | --- |
| Site in Csd | *B.ter.* | *B.imp.* | *A.cer.* | *A.dor.* | *A.flo.* | *A.mel.* |
| /Fem1 tree | **T** | **T** | **T** | **I** | **T** | **T** |

| **Node 21 – Fem** | **Node 22 – Fem** | **Node17 – Csd / Fem1** |
| --- | --- | --- |
| **E** | **D** | **E** |

| Position in Fem protein sequence alignment: # 79 | | | | | | | |
| --- | --- | --- | --- | --- | --- | --- | --- |
|  | *C.flo.* | *A.cep.* | *H.sal.* | *A.ech.* | *S.inv.* | *P.bar.* | *N.vit.* |
| Site in | **E** | **D** | **D** | **D** | **D** | **D** | **D** |
| Fem tree | *B.ter.* | *B.imp.* | *A.cer.* | *A.dor.* | *A.flo.* | *A.mel.* | *M.com.* |
|  | **E** | **E** | **E** | **E** | **E** | **E** | **E** |

| Position in Csd/Fem1 protein sequence alignment: # 74 | | | | | | |
| --- | --- | --- | --- | --- | --- | --- |
| Site in Csd | *B.ter.* | *B.imp.* | *A.cer.* | *A.dor.* | *A.flo.* | *A.mel.* |
| /Fem1 tree | **E** | **E** | **K** | **E** | **K** | **E** |

| **Node 21 – Fem** | **Node 22 – Fem** | **Node17 – Csd / Fem1** |
| --- | --- | --- |
| **I** | **V** | **I** |

| Position in Fem protein sequence alignment: # 119 | | | | | | | |
| --- | --- | --- | --- | --- | --- | --- | --- |
|  | *C.flo.* | *A.cep.* | *H.sal.* | *A.ech.* | *S.inv.* | *P.bar.* | *N.vit.* |
| Site in | **V** | **L** | **L** | **L** | **L** | **L** | **V** |
| Fem tree | *B.ter.* | *B.imp.* | *A.cer.* | *A.dor.* | *A.flo.* | *A.mel.* | *M.com.* |
|  | **I** | **I** | **I** | **I** | **I** | **I** | **V** |

| Position in Csd/Fem1 protein sequence alignment: # 116 | | | | | | |
| --- | --- | --- | --- | --- | --- | --- |
| Site in Csd | *B.ter.* | *B.imp.* | *A.cer.* | *A.dor.* | *A.flo.* | *A.mel.* |
| /Fem1 tree | **I** | **I** | **I** | **I** | **I** | **I** |

| **Node 21 – Fem** | **Node 22 – Fem** | **Node17 – Csd / Fem1** |
| --- | --- | --- |
| **E** | **D** | **E** |

| Position in Fem protein sequence alignment: # 135 | | | | | | | |
| --- | --- | --- | --- | --- | --- | --- | --- |
|  | *C.flo.* | *A.cep.* | *H.sal.* | *A.ech.* | *S.inv.* | *P.bar.* | *N.vit.* |
| Site in | **D** | **D** | **E** | **D** | **D** | **D** | **D** |
| Fem tree | *B.ter.* | *B.imp.* | *A.cer.* | *A.dor.* | *A.flo.* | *A.mel.* | *M.com.* |
|  | **E** | **E** | **E** | **E** | **E** | **E** | **E** |

| Position in Csd/Fem1 protein sequence alignment: # 130 | | | | | | |
| --- | --- | --- | --- | --- | --- | --- |
| Site in Csd | *B.ter.* | *B.imp.* | *A.cer.* | *A.dor.* | *A.flo.* | *A.mel.* |
| /Fem1 tree | **E** | **E** | **E** | **E** | **E** | **E** |

| **Node 21 – Fem** | **Node 22 – Fem** | **Node17 – Csd / Fem1** |
| --- | --- | --- |
| **E** | **D** | **E** |

| Position in Fem protein sequence alignment: # 240 | | | | | | | |
| --- | --- | --- | --- | --- | --- | --- | --- |
|  | *C.flo.* | *A.cep.* | *H.sal.* | *A.ech.* | *S.inv.* | *P.bar.* | *N.vit.* |
| Site in | **D** | **D** | **D** | **D** | **D** | **E** | **D** |
| Fem tree | *B.ter.* | *B.imp.* | *A.cer.* | *A.dor.* | *A.flo.* | *A.mel.* | *M.com.* |
|  | **E** | **E** | **E** | **E** | **E** | **E** | **E** |

| Position in Csd/Fem1 protein sequence alignment: # 235 | | | | | | |
| --- | --- | --- | --- | --- | --- | --- |
| Site in Csd | *B.ter.* | *B.imp.* | *A.cer.* | *A.dor.* | *A.flo.* | *A.mel.* |
| /Fem1 tree | **E** | **E** | **E** | **E** | **E** | **E** |

| **Node 21 – Fem** | **Node 22 – Fem** | **Node17 – Csd / Fem1** |
| --- | --- | --- |
| **V** | **I** | **V** |

| Position in Fem protein sequence alignment: # 264 | | | | | | | |
| --- | --- | --- | --- | --- | --- | --- | --- |
|  | *C.flo.* | *A.cep.* | *H.sal.* | *A.ech.* | *S.inv.* | *P.bar.* | *N.vit.* |
| Site in | **I** | **I** | **I** | **I** | **I** | **I** | **I** |
| Fem tree | *B.ter.* | *B.imp.* | *A.cer.* | *A.dor.* | *A.flo.* | *A.mel.* | *M.com.* |
|  | **V** | **V** | **V** | **V** | **V** | **V** | **F** |

| Position in Csd/Fem1 protein sequence alignment: # 263 | | | | | | |
| --- | --- | --- | --- | --- | --- | --- |
| Site in Csd | *B.ter.* | *B.imp.* | *A.cer.* | *A.dor.* | *A.flo.* | *A.mel.* |
| /Fem1 tree | **V** | **V** | **V** | **V** | **V** | **V** |

| **Node 21 – Fem** | **Node 22 – Fem** | **Node17 – Csd / Fem1** |
| --- | --- | --- |
| **M** | **L** | **M** |

| Position in Fem protein sequence alignment: # 290 | | | | | | | |
| --- | --- | --- | --- | --- | --- | --- | --- |
|  | *C.flo.* | *A.cep.* | *H.sal.* | *A.ech.* | *S.inv.* | *P.bar.* | *N.vit.* |
| Site in | **L** | **L** | **L** | **L** | **L** | **L** | **L** |
| Fem tree | *B.ter.* | *B.imp.* | *A.cer.* | *A.dor.* | *A.flo.* | *A.mel.* | *M.com.* |
|  | **M** | **M** | **I** | **I** | **I** | **I** | **M** |

| Position in Csd/Fem1 protein sequence alignment: # 287 | | | | | | |
| --- | --- | --- | --- | --- | --- | --- |
| Site in Csd | *B.ter.* | *B.imp.* | *A.cer.* | *A.dor.* | *A.flo.* | *A.mel.* |
| /Fem1 tree | **M** | **M** | **I** | **I** | **I** | **I** |
