# Supplementary material for: Independent Evolutionary Origin of fem Paralogous Genes and Complementary Sex Determination in Hymenopteran Insects
Source: PLoS One. 2014 Apr 17;9(4):e91883. doi: 10.1371/journal.pone.0091883 (PMC3990544; doi:10.1371/journal.pone.0091883)
Supplement: Figure S5 — The informative substitutions found in the Bombus lineage (Fem tree) that were used in Figure 3 . The identity of the different species and nodes of the Fem and Fem1 protein tree is shown. Site number (#) indicates the position in the alignment of the Fem and of the Csd/Fem1 protein sequences. (DOCX) [file pone.0091883.s005.docx]

**Figure S5**. The informative substitutions found in the *Bombus* lineage (Fem tree) that were used in Figure 3. The identity of the different species and nodes of the Fem and Fem1 protein tree is shown. Site number (#) indicates the position in the alignment of the Fem and of the Csd/Fem1 protein sequences.

| **Node 19**-**Fem** |
| --- |
| **L** |

| Position in Fem protein sequence alignment: # 48 | | | | | |
| --- | --- | --- | --- | --- | --- |
|  | Node 12 (*B.imp.*)-Fem | | Node 1 (*B.ter.*)-Fem | | |
| Site in | **I** | | **L** | | |
| Fem tree | *A.mel.*-Fem | *A.dor.*-Fem | *A.cer.*-Fem | *A.flo*-Fem | *M.com.*-Fem |
|  | **R** | **R** | **R** | **R** | **R** |

| Position in Csd/Fem1 protein sequence alignment: # 44 | | |
| --- | --- | --- |
| Site in Csd | Node 11 (*B.imp*)-Fem 1 | Node 1 (*B.ter*)-Fem 1 |
| /Fem1 tree | **L** | **L** |

| **Node 19**-**Fem** |
| --- |
| **V** |

| Position in Fem protein sequence alignment: # 146 | | | | | |
| --- | --- | --- | --- | --- | --- |
|  | Node 12 (*B.imp.*)-Fem | | Node 1 (*B.ter.*)-Fem | | |
| Site in | **E** | | **V** | | |
| Fem tree | *A.mel.*-Fem | *A.dor.*-Fem | *A.cer.*-Fem | *A.flo*-Fem | *M.com.*-Fem |
|  | **T** | **T** | **T** | **T** | **V** |

| Position in Csd/Fem1 protein sequence alignment: # 141 | | |
| --- | --- | --- |
| Site in Csd | Node 11 (*B.imp*)-Fem 1 | Node 1 (*B.ter*)-Fem 1 |
| /Fem1 tree | **V** | **V** |

| **Node 19**-**Fem** |
| --- |
| **V** |

| Position in Fem protein sequence alignment: # 164 | | | | | |
| --- | --- | --- | --- | --- | --- |
|  | Node 12 (*B.imp.*)-Fem | | Node 1 (*B.ter.*)-Fem | | |
| Site in | **M** | | **V** | | |
| Fem tree | *A.mel.*-Fem | *A.dor.*-Fem | *A.cer.*-Fem | *A.flo*-Fem | *M.com.*-Fem |
|  | **T** | **T** | **T** | **T** | **V** |

| Site was deleted in the Fem tree analysis due to gaps (site # 244 in the overall alignment) | | |
| --- | --- | --- |
| Site in Csd | Node 11 (*B.imp*)-Fem 1 | Node 1 (*B.ter*)-Fem 1 |
| /Fem1 tree | **A** | **A** |

| **Node 19**-**Fem** |
| --- |
| **G** |

| Position in Fem protein sequence alignment: # 201 | | | | | |
| --- | --- | --- | --- | --- | --- |
|  | Node 12 (*B.imp.*)-Fem | | Node 1 (*B.ter.*)-Fem | | |
| Site in | **G** | | **D** | | |
| Fem tree | *A.mel.*-Fem | *A.dor.*-Fem | *A.cer.*-Fem | *A.flo*-Fem | *M.com.*-Fem |
|  | **D** | **D** | **D** | **D** | **G** |

| Position in Csd/Fem1 protein sequence alignment: # 186 | | |
| --- | --- | --- |
| Site in Csd | Node 11 (*B.imp*)-Fem 1 | Node 1 (*B.ter*)-Fem 1 |
| /Fem1 tree | **G** | **G** |

| **Node 19**-**Fem** |
| --- |
| **R** |

| Position in Fem protein sequence alignment: # 210 | | | | | |
| --- | --- | --- | --- | --- | --- |
|  | Node 12 (*B.imp.*)-Fem | | Node 1 (*B.ter.*)-Fem | | |
| Site in | **R** | | **K** | | |
| Fem tree | *A.mel.*-Fem | *A.dor.*-Fem | *A.cer.*-Fem | *A.flo*-Fem | *M.com.*-Fem |
|  | **R** | **R** | **R** | **R** | **R** |

| Position in Csd/Fem1 protein sequence alignment: # 195 | | |
| --- | --- | --- |
| Site in Csd | Node 11 (*B.imp*)-Fem 1 | Node 1 (*B.ter*)-Fem 1 |
| /Fem1 tree | **R** | **R** |

| **Node 19**-**Fem** |
| --- |
| **M** |

| Position in Fem protein sequence alignment: # 291 | | | | | |
| --- | --- | --- | --- | --- | --- |
|  | Node 12 (*B.imp.*)-Fem | | Node 1 (*B.ter.*)-Fem | | |
| Site in | **I** | | **M** | | |
| Fem tree | *A.mel.*-Fem | *A.dor.*-Fem | *A.cer.*-Fem | *A.flo*-Fem | *M.com.*-Fem |
|  | **G** | **G** | **G** | **G** | **M** |

| Position in Csd/Fem1 protein sequence alignment: # 288 | | |
| --- | --- | --- |
| Site in Csd | Node 11 (*B.imp*)-Fem 1 | Node 1 (*B.ter*)-Fem 1 |
| /Fem1 tree | **M** | **M** |
