# Supplementary material for: Independent Evolutionary Origin of fem Paralogous Genes and Complementary Sex Determination in Hymenopteran Insects
Source: PLoS One. 2014 Apr 17;9(4):e91883. doi: 10.1371/journal.pone.0091883 (PMC3990544; doi:10.1371/journal.pone.0091883)
Supplement: Figure S6 — The informative substitutions found in the Bombus lineage (Fem1 tree) that were used in Figure 3 . The identity of the different species and nodes of the Fem and Fem1 protein tree is shown. Site number (#) indicate the position in the alignment of the Fem and of the Csd/Fem1 protein sequences. (DOCX) [file pone.0091883.s006.docx]

**Figure S6:**The informative substitutions found in the *Bombus* lineage (Fem1 tree) that were used in Figure 3. The identity of the different species and nodes of the Fem and Fem1 protein tree is shown. Site number (#) indicate the position in the alignment of the Fem and of the Csd/Fem1 protein sequences.

| **Node 16**-**Fem1** |
| --- |
| **R** |

| Position in Csd/Fem1 protein sequence alignment: # 17 | | | | |
| --- | --- | --- | --- | --- |
|  | Node 11 (*B.imp.*)-Fem1 | | Node 1 (*B.ter.*) | |
| Site in Csd | **R** | | **K** | |
| /Fem1 tree | *A.mel.*-Csd | *A.dor.*-Csd | *A.cer.*-Csd | *A.flo.*-Csd |
|  | **R** | **K** | **R** | **R** |

| Position in Fem protein sequence alignment: # 20 | | |
| --- | --- | --- |
| Site in Fem | Node 11 (*B.imp*)-Fem 1 | Node 1 (*B.ter*)-Fem 1 |
| tree | **R** | **R** |

| **Node 16**-**Fem1** |
| --- |
| **I** |

| Position in Csd/Fem1 protein sequence alignment: # 22 | | | | |
| --- | --- | --- | --- | --- |
|  | Node 11 (*B.imp.*)-Fem1 | | Node 1 (*B.ter.*) | |
| Site in Csd | **V** | | **I** | |
| /Fem1 tree | *A.mel.*-Csd | *A.dor.*-Csd | *A.cer.*-Csd | *A.flo.*-Csd |
|  | **V** | **I** | **I** | **I** |

| Position in Fem protein sequence alignment: # 25 | | |
| --- | --- | --- |
| Site in Fem | Node 11 (*B.imp*)-Fem 1 | Node 1 (*B.ter*)-Fem 1 |
| tree | **I** | **I** |

| **Node 16**-**Fem1** |
| --- |
| **K** |

| Position in Csd/Fem1 protein sequence alignment: # 32 | | | | |
| --- | --- | --- | --- | --- |
|  | Node 11 (*B.imp.*)-Fem1 | | Node 1 (*B.ter.*) | |
| Site in Csd | **K** | | **R** | |
| /Fem1 tree | *A.mel.*-Csd | *A.dor.*-Csd | *A.cer.*-Csd | *A.flo.*-Csd |
|  | **K** | **K** | **K** | **R** |

| Position in Fem protein sequence alignment: # 35 | | |
| --- | --- | --- |
| Site in Fem | Node 11 (*B.imp*)-Fem 1 | Node 1 (*B.ter*)-Fem 1 |
| tree | **K** | **K** |

| **Node 16**-**Fem1** |
| --- |
| **K** |

| Position in Csd/Fem1 protein sequence alignment: # 52 | | | | |
| --- | --- | --- | --- | --- |
|  | Node 11 (*B.imp.*)-Fem1 | | Node 1 (*B.ter.*) | |
| Site in Csd | **T** | | **K** | |
| /Fem1 tree | *A.mel.*-Csd | *A.dor.*-Csd | *A.cer.*-Csd | *A.flo.*-Csd |
|  | **K** | **K** | **K** | **K** |

| Position in Fem protein sequence alignment: # 57 | | |
| --- | --- | --- |
| Site in Fem | Node 11 (*B.imp*)-Fem 1 | Node 1 (*B.ter*)-Fem 1 |
| tree | **K** | **K** |

| **Node 16**-**Fem1** |
| --- |
| **S** |

| Position in Csd/Fem1 protein sequence alignment: # 53 | | | | |
| --- | --- | --- | --- | --- |
|  | Node 11 (*B.imp.*)-Fem1 | | Node 1 (*B.ter.*) | |
| Site in Csd | **S** | | **N** | |
| /Fem1 tree | *A.mel.*-Csd | *A.dor.*-Csd | *A.cer.*-Csd | *A.flo.*-Csd |
|  | **T** | **T** | **T** | **T** |

| Position in Fem protein sequence alignment: # 58 | | |
| --- | --- | --- |
| Site in Fem | Node 11 (*B.imp*)-Fem 1 | Node 1 (*B.ter*)-Fem 1 |
| tree | **S** | **S** |

| **Node 16**-**Fem1** |
| --- |
| **K** |

| Position in Csd/Fem1 protein sequence alignment: # 54 | | | | |
| --- | --- | --- | --- | --- |
|  | Node 11 (*B.imp.*)-Fem1 | | Node 1 (*B.ter.*) | |
| Site in Csd | **E** | | **K** | |
| /Fem1 tree | *A.mel.*-Csd | *A.dor.*-Csd | *A.cer.*-Csd | *A.flo.*-Csd |
|  | **K** | **K** | **K** | **K** |

| Position in Fem protein sequence alignment: # 59 | | |
| --- | --- | --- |
| Site in Fem | Node 11 (*B.imp*)-Fem 1 | Node 1 (*B.ter*)-Fem 1 |
| tree | **K** | **K** |

| **Node 16**-**Fem1** |
| --- |
| **H** |

| Position in Csd/Fem1 protein sequence alignment: # 62 | | | | |
| --- | --- | --- | --- | --- |
|  | Node 11 (*B.imp.*)-Fem1 | | Node 1 (*B.ter.*) | |
| Site in Csd | **H** | | **Q** | |
| /Fem1 tree | *A.mel.*-Csd | *A.dor.*-Csd | *A.cer.*-Csd | *A.flo.*-Csd |
|  | **D** | **D** | **D** | **K** |

| Position in Fem protein sequence alignment: # 64 | | |
| --- | --- | --- |
| Site in Fem | Node 11 (*B.imp*)-Fem 1 | Node 1 (*B.ter*)-Fem 1 |
| tree | **H** | **H** |

| **Node 16**-**Fem1** |
| --- |
| **S** |

| Position in Csd/Fem1 protein sequence alignment: # 64 | | | | |
| --- | --- | --- | --- | --- |
|  | Node 11 (*B.imp.*)-Fem1 | | Node 1 (*B.ter.*) | |
| Site in Csd | **L** | | **S** | |
| /Fem1 tree | *A.mel.*-Csd | *A.dor.*-Csd | *A.cer.*-Csd | *A.flo.*-Csd |
|  | **S** | **S** | **S** | **P** |

| Position in Fem protein sequence alignment: # 69 | | |
| --- | --- | --- |
| Site in Fem | Node 11 (*B.imp*)-Fem 1 | Node 1 (*B.ter*)-Fem 1 |
| tree | **S** | **S** |

| **Node 16**-**Fem1** |
| --- |
| **N** |

| Position in Csd/Fem1 protein sequence alignment: # 65 | | | | |
| --- | --- | --- | --- | --- |
|  | Node 11 (*B.imp.*)-Fem1 | | Node 1 (*B.ter.*) | |
| Site in Csd | **S** | | **N** | |
| /Fem1 tree | *A.mel.*-Csd | *A.dor.*-Csd | *A.cer.*-Csd | *A.flo.*-Csd |
|  | **N** | **N** | **N** | **N** |

| Position in Fem protein sequence alignment: # 70 | | |
| --- | --- | --- |
| Site in Fem | Node 11 (*B.imp*)-Fem 1 | Node 1 (*B.ter*)-Fem 1 |
| tree | **N** | **N** |

| **Node 16**-**Fem1** |
| --- |
| **P** |

| Position in Csd/Fem1 protein sequence alignment: # 85 | | | | |
| --- | --- | --- | --- | --- |
|  | Node 11 (*B.imp.*)-Fem1 | | Node 1 (*B.ter.*) | |
| Site in Csd | **P** | | **L** | |
| /Fem1 tree | *A.mel.*-Csd | *A.dor.*-Csd | *A.cer.*-Csd | *A.flo.*-Csd |
|  | **P** | **S** | **P** | **P** |

| Position in Fem protein sequence alignment: # 90 | | |
| --- | --- | --- |
| Site in Fem | Node 11 (*B.imp*)-Fem 1 | Node 1 (*B.ter*)-Fem 1 |
| tree | **P** | **P** |

| **Node 16**-**Fem1** |
| --- |
| **V** |

| Position in Csd/Fem1 protein sequence alignment: # 137 | | | | |
| --- | --- | --- | --- | --- |
|  | Node 11 (*B.imp.*)-Fem1 | | Node 1 (*B.ter.*) | |
| Site in Csd | **V** | | **A** | |
| /Fem1 tree | *A.mel.*-Csd | *A.dor.*-Csd | *A.cer.*-Csd | *A.flo.*-Csd |
|  | **V** | **V** | **V** | **V** |

| Position in Fem protein sequence alignment: # 142 | | |
| --- | --- | --- |
| Site in Fem | Node 11 (*B.imp*)-Fem 1 | Node 1 (*B.ter*)-Fem 1 |
| tree | **T** | **T** |

| **Node 16**-**Fem1** |
| --- |
| **T** |

| Position in Csd/Fem1 protein sequence alignment: # 138 | | | | |
| --- | --- | --- | --- | --- |
|  | Node 11 (*B.imp.*)-Fem1 | | Node 1 (*B.ter.*) | |
| Site in Csd | **T** | | **R** | |
| /Fem1 tree | *A.mel.*-Csd | *A.dor.*-Csd | *A.cer.*-Csd | *A.flo.*-Csd |
|  | **L** | **L** | **F** | **L** |

| Position in Fem protein sequence alignment: # 143 | | |
| --- | --- | --- |
| Site in Fem | Node 11 (*B.imp*)-Fem 1 | Node 1 (*B.ter*)-Fem 1 |
| tree | **T** | **T** |

| **Node 16**-**Fem1** |
| --- |
| **K** |

| Position in Csd/Fem1 protein sequence alignment: # 158 | | | | |
| --- | --- | --- | --- | --- |
|  | Node 11 (*B.imp.*)-Fem1 | | Node 1 (*B.ter.*) | |
| Site in Csd | **E** | | **K** | |
| /Fem1 tree | *A.mel.*-Csd | *A.dor.*-Csd | *A.cer.*-Csd | *A.flo.*-Csd |
|  | **K** | **N** | **K** | **k** |

| Position in Fem protein sequence alignment: # 167 | | |
| --- | --- | --- |
| Site in Fem | Node 11 (*B.imp*)-Fem 1 | Node 1 (*B.ter*)-Fem 1 |
| tree | **K** | **K** |

| **Node 16**-**Fem1** |
| --- |
| **E** |

| Position in Csd/Fem1 protein sequence alignment: # 218 | | | | |
| --- | --- | --- | --- | --- |
|  | Node 11 (*B.imp.*)-Fem1 | | Node 1 (*B.ter.*) | |
| Site in Csd | **G** | | **E** | |
| /Fem1 tree | *A.mel.*-Csd | *A.dor.*-Csd | *A.cer.*-Csd | *A.flo.*-Csd |
|  | **E** | **E** | **E** | **E** |

| Position in Fem protein sequence alignment: # 227 | | |
| --- | --- | --- |
| Site in Fem | Node 11 (*B.imp*)-Fem 1 | Node 1 (*B.ter*)-Fem 1 |
| tree | **E** | **E** |

| **Node 16**-**Fem1** |
| --- |
| **R** |

| Position in Csd/Fem1 protein sequence alignment: # 247 | | | | |
| --- | --- | --- | --- | --- |
|  | Node 11 (*B.imp.*)-Fem1 | | Node 1 (*B.ter.*) | |
| Site in Csd | **R** | | **G** | |
| /Fem1 tree | *A.mel.*-Csd | *A.dor.*-Csd | *A.cer.*-Csd | *A.flo.*-Csd |
|  | **K** | **R** | **R** | **R** |

| Position in Fem protein sequence alignment: # 249 | | |
| --- | --- | --- |
| Site in Fem | Node 11 (*B.imp*)-Fem 1 | Node 1 (*B.ter*)-Fem 1 |
| tree | **R** | **R** |

| **Node 16**-**Fem1** |
| --- |
| **R** |

| Position in Csd/Fem1 protein sequence alignment: # 250 | | | | |
| --- | --- | --- | --- | --- |
|  | Node 11 (*B.imp.*)-Fem1 | | Node 1 (*B.ter.*) | |
| Site in Csd | **R** | | **T** | |
| /Fem1 tree | *A.mel.*-Csd | *A.dor.*-Csd | *A.cer.*-Csd | *A.flo.*-Csd |
|  | **K** | **R** | **K** | **T** |

| Position in Fem protein sequence alignment: # 251 | | |
| --- | --- | --- |
| Site in Fem | Node 11 (*B.imp*)-Fem 1 | Node 1 (*B.ter*)-Fem 1 |
| tree | **R** | **R** |

| **Node 16**-**Fem1** |
| --- |
| **P** |

| Position in Csd/Fem1 protein sequence alignment: # 274 | | | | |
| --- | --- | --- | --- | --- |
|  | Node 11 (*B.imp.*)-Fem1 | | Node 1 (*B.ter.*) | |
| Site in Csd | **P** | | **H** | |
| /Fem1 tree | *A.mel.*-Csd | *A.dor.*-Csd | *A.cer.*-Csd | *A.flo.*-Csd |
|  | **P** | **P** | **P** | **S** |

| Position in Fem protein sequence alignment: # 277 | | |
| --- | --- | --- |
| Site in Fem | Node 11 (*B.imp*)-Fem 1 | Node 1 (*B.ter*)-Fem 1 |
| tree | **P** | **P** |

| **Node 16**-**Fem1** |
| --- |
| **P** |

| Position in Csd/Fem1 protein sequence alignment: # 300 | | | | |
| --- | --- | --- | --- | --- |
|  | Node 11 (*B.imp.*)-Fem1 | | Node 1 (*B.ter.*) | |
| Site in Csd | **P** | | **S** | |
| /Fem1 tree | *A.mel.*-Csd | *A.dor.*-Csd | *A.cer.*-Csd | *A.flo.*-Csd |
|  | **P** | **P** | **P** | **P** |

| Position in Fem protein sequence alignment: # 309 | | |
| --- | --- | --- |
| Site in Fem | Node 11 (*B.imp*)-Fem 1 | Node 1 (*B.ter*)-Fem 1 |
| tree | **P** | **P** |

| **Node 16**-**Fem1** |
| --- |
| **L** |

| Position in Csd/Fem1 protein sequence alignment: # 301 | | | | |
| --- | --- | --- | --- | --- |
|  | Node 11 (*B.imp.*)-Fem1 | | Node 1 (*B.ter.*) | |
| Site in Csd | **F** | | **L** | |
| /Fem1 tree | *A.mel.*-Csd | *A.dor.*-Csd | *A.cer.*-Csd | *A.flo.*-Csd |
|  | **P** | **P** | **P** | **P** |

| Position in Fem protein sequence alignment: # 310 | | |
| --- | --- | --- |
| Site in Fem | Node 11 (*B.imp*)-Fem 1 | Node 1 (*B.ter*)-Fem 1 |
| tree | **P** | **P** |
