# Supplementary material for: Independent Evolutionary Origin of fem Paralogous Genes and Complementary Sex Determination in Hymenopteran Insects
Source: PLoS One. 2014 Apr 17;9(4):e91883. doi: 10.1371/journal.pone.0091883 (PMC3990544; doi:10.1371/journal.pone.0091883)
Supplement: Figure S7 — The number of falsely detected events (FDE) using the methods as indicated on the X axis. These programs were implemented in the RDP 3.44 software program. The methods were run on each of the 20 alignments which consisted of two randomly chosen csd sequences from a single Apis species and one fem sequence from B. terrestris. These events are falsely detected as this transfer involve the outgroup Bombus sequence and the polymorphism between csd alleles which newly evolved in the different Apis species. (DOCX) [file pone.0091883.s007.docx]

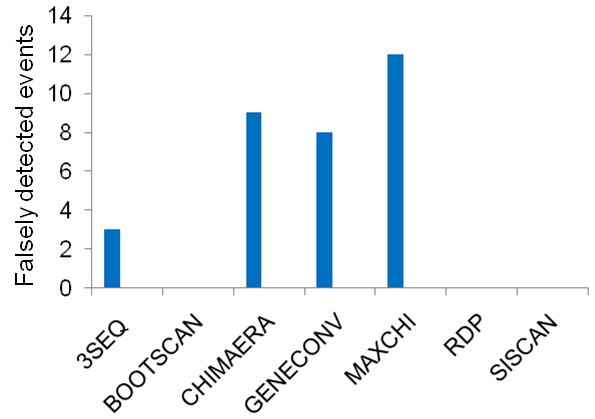


**Figure S7:** The number of falsely detected events (FDE) using the methods as indicated on the X axis. These programs were implemented in the RDP 3.44 software program. The methods were run on each of the 20 alignments which consisted of two randomly chosen *csd* sequences from a single *Apis* species and one *fem* sequence from *B*. *terrestris*.These events are falsely detected as this transfer involve the outgroup *Bombus* sequence and the polymorphism between csd alleles which newly evolved in the different *Apis* species.
