# Supplementary material for: Independent Evolutionary Origin of fem Paralogous Genes and Complementary Sex Determination in Hymenopteran Insects
Source: PLoS One. 2014 Apr 17;9(4):e91883. doi: 10.1371/journal.pone.0091883 (PMC3990544; doi:10.1371/journal.pone.0091883)
Supplement: Table S1 — The dS i values for the paralogous gene pairs fem and csd/fem1 within each species (Ador, Apis dorsata; Amel, Apis mellifera; Bimp, Bombus impatiens; Bter, Bombus terrestris). (DOCX) [file pone.0091883.s008.docx]

**Table S1**. The *d_S i_* values for the paralogous gene pairs *fem* and *csd*/*fem*1 within each species

(*Ador, Apis dorsata*; *Amel*, *Apis mellifera*; *Bimp, Bombus impatiens*; *Bter*, *Bombus terrestris*).

| Species | *d_S i_ between fem* and *csd/fem*1 |
| --- | --- |
| A.mel | 0.18 |
| A.dor | 0.18 |
| B.ter | 0.24 |
| B.imp | 0.22 |
